# VOLTA: adVanced mOLecular neTwork Analysis

**DOI:** 10.1093/bioinformatics/btab642

**Published:** 2021-09-08

**Authors:** Alisa Pavel, Antonio Federico, Giusy del Giudice, Angela Serra, Dario Greco

**Affiliations:** Faculty of Medicine and Health Technology, Tampere University, Tampere 33520, Finland; BioMediTech Institute, Tampere University, Tampere 33520, Finland; Finnish Hub for Development and Validation of Integrated Approaches (FHAIVE), Faculty of Medicine and Health Technology, Tampere University, Tampere 33520, Finland; Faculty of Medicine and Health Technology, Tampere University, Tampere 33520, Finland; BioMediTech Institute, Tampere University, Tampere 33520, Finland; Finnish Hub for Development and Validation of Integrated Approaches (FHAIVE), Faculty of Medicine and Health Technology, Tampere University, Tampere 33520, Finland; Faculty of Medicine and Health Technology, Tampere University, Tampere 33520, Finland; BioMediTech Institute, Tampere University, Tampere 33520, Finland; Finnish Hub for Development and Validation of Integrated Approaches (FHAIVE), Faculty of Medicine and Health Technology, Tampere University, Tampere 33520, Finland; Faculty of Medicine and Health Technology, Tampere University, Tampere 33520, Finland; BioMediTech Institute, Tampere University, Tampere 33520, Finland; Finnish Hub for Development and Validation of Integrated Approaches (FHAIVE), Faculty of Medicine and Health Technology, Tampere University, Tampere 33520, Finland; Faculty of Medicine and Health Technology, Tampere University, Tampere 33520, Finland; BioMediTech Institute, Tampere University, Tampere 33520, Finland; Finnish Hub for Development and Validation of Integrated Approaches (FHAIVE), Faculty of Medicine and Health Technology, Tampere University, Tampere 33520, Finland; Institute of Biotechnology, University of Helsinki, Helsinki 00790, Finland

## Abstract

**Motivation:**

Network analysis is a powerful approach to investigate biological systems. It is often applied to study gene co-expression patterns derived from transcriptomics experiments. Even though co-expression analysis is widely used, there is still a lack of tools that are open and customizable on the basis of different network types and analysis scenarios (e.g. through function accessibility), but are also suitable for novice users by providing complete analysis pipelines.

**Results:**

We developed VOLTA, a Python package suited for complex co-expression network analysis. VOLTA is designed to allow users direct access to the individual functions, while they are also provided with complete analysis pipelines. Moreover, VOLTA offers when possible multiple algorithms applicable to each analytical step (e.g. multiple community detection or clustering algorithms are provided), hence providing the user with the possibility to perform analysis tailored to their needs. This makes VOLTA highly suitable for experienced users who wish to build their own analysis pipelines for a wide range of networks as well as for novice users for which a ‘plug and play’ system is provided.

**Availability and implementation:**

The package and used data are available at GitHub: https://github.com/fhaive/VOLTA and 10.5281/zenodo.5171719.

**Supplementary information:**

Supplementary data are available at *Bioinformatics* online.

## 1 Introduction

Co-expression network analysis has become popular to characterize gene–gene expression patterns from omics data by providing insight into the differential gene co-expression patterns and their local and global organizations, between different biological conditions (van Dam *et al.*, 2018; Liu *et al.*, 2017). Currently three main classes of network analysis software exist to (i) infer co-expression networks from experimental data (Marwah *et al.*, 2018), (ii) investigate the properties of individual networks (Hagberg *et al.*, 2008) and (iii) compare multiple networks (Proost and Mutwil, 2018), while flexible, comprehensive tools are currently still missing. We therefore developed VOLTA, a Python package that combines traditional network metrics with functions adjusted to the comparison and evaluation of co-expression networks. In addition, VOLTA is highly versatile by nature, allowing users easy access to all functionalities and parameters. This helps the users to create analytical pipelines to answer a wide range of biological questions, which is in contrast to many other available software/tools which are restricted to specific steps through their implementation (Proost and Mutwil, 2018; Supplementary Text). To the best of our knowledge there is currently no other package available, which combines a diverse set of network analysis methods into a single package, completely exposes its internal functionalities and therefore is highly versatile.

## 2 Implementation

VOLTA consists of seven modules (Supplementary Fig. S2), which can be used independently or in combination to create complex analytical pipelines. VOLTA is implemented in Python 3 and allows users deep access to all functionalities and parameter settings. In addition to the main function modules, VOLTA provides six predefined pipeline wrappers. Three fully functional pipelines are provided in the form of Jupyter Notebooks respectively addressing: (i) *clustering of multiple networks* employing global and local similarities; (ii) *identification of common connectivity patterns in a set of networks* and (iii) *network–network comparison* based on their nodes, edges and communities (Supplementary Text S1).

## 3 Application

To demonstrate the functionalities and applicability of VOLTA in co-expression network analysis, we selected three possible analysis scenarios. The networks for this study were generated from the Lincs 1000 data (Supplementary Text S4.1). In the first case, in order to describe the transcriptional perturbation induced on A549 cells by treatment with dasatinib and mitoxantrone, we compared the characteristics (i.e. connectivity) of the two co-expression networks by exploiting the functionalities of the VOLTA package. Such an analysis allowed the characterization of the specific mechanism of action of the considered chemotherapeutic drugs. Evaluation of difference in gene centrality in the two networks, showcases a high difference in centrality among the networks of *OXA1L*, *YME1L1 and DNAJC15* genes, suggesting an involvement of mitoxantrone in the impairment of mitochondrial function, as has been previously demonstrated (Rossato *et al.*, 2014). Comparison of pathway enrichment of the modules of the two networks showcases the difference in mechanisms of mitoxantrone and dasatinib. Modules detected in the mitoxantrone network enrich for DNA double strand break pathways, highlighting the genotoxic effect of mitoxantrone. On the other hand, functional characterization of the modules in the dasatinib network highlight the involvement in the intracellular signaling processes (Supplementary Text S4.2).

In the second case study, we aimed to assess the impact of the different molecular makeup of 20 cancer cell lines on the mechanism of action of dasatinib (Supplementary Text S4). Exploiting VOLTA functionalities for this aim allowed the investigation of drug sensitivity profiles of cancer cell lines to dasatinib treatment and to identify clusters of similarly responding cell lines. The three clusters that could be identified were (i) a cluster mainly made up of breast (cancer) related tissues, (ii) one containing ‘normal’ samples from different tissues and (iii) another one containing different tissue types—not fitting into the previous two clusters (Supplementary Table S8). In the third analysis, we showcased and characterized the statistical sub-graph of the breast related tissue cluster. Investigation of the cluster characterized sub-graph reveals genes that are involved in processes related to cell cycle, differentiation and metabolism as central. Pathway enrichment of the modules of the characterized sub-graph indicates a deregulation of immune-related pathways, together with cell cycle and DNA repair machinery (Supplementary Table S10).

## 4 Discussion

To date, many network analysis software solutions have been proposed, which have often either very general purpose (Hagberg *et al.*, 2008) or they are specialized packages to solve a specific problem (Rossetti *et al.*, 2019). Software solutions for co-expression network analysis, on the other hand, are commonly optimized for a single analysis pipeline or step (Marwah *et al.*, 2018; Proost and Mutwil, 2018). While these tools are easy to use, they can have the downside of being non-adaptable to other problems. This can for example result through stringent input format requirements, or commonly that individual functionalities are implemented in such a way that they are not accessible from outside the provided software, which often means that individual functionalities (of a pipeline) cannot be re-used outside the ‘intended’ flow as well as that parameter adjustment is restricted (Supplementary Text S2). We therefore developed VOLTA, which combines a diverse set of exposed functions, applicable in many different fields of network analysis and aims, when possible, to provide different algorithms for a given task (for example a diverse set of community detection algorithms is provided). This allows users to customize their pipelines, for example based on their network structure, or allows the application of ensemble methods. In addition, pipelines (which can easily be modified by users due to being provided as Jupyter Notebook files (https://github.com/fhaive/VOLTA/tree/master/jupyternotebooks) for specific analysis in the domain of co-expression networks are provided. This allows inexperienced users a plug-and-play experience, while more advanced users have the possibility to construct customized pipelines.

## 5 Conclusion

Here, we presented VOLTA, a Python package highly adapted to biological network analysis (with a focus on co-expression networks). It is the first package providing a wide range of functionalities adaptable to different studies in Python, which is both suited to naive as well as expert users. The usability and applicability of VOLTA in (co-expression) network analysis has been highlighted in the performed case studies.

## Supplementary Material

btab642_Supplementary_Data
