# Supplementary material for: VOLTA: adVanced mOLecular neTwork Analysis
Source: Bioinformatics. 2021 Sep 8;37(23):4587–8. doi: 10.1093/bioinformatics/btab642 (PMC8687180; doi:10.1093/bioinformatics/btab642)
Supplement: btab642_Supplementary_Data [file btab642_supplementary_data.pdf]

VOLTA: adVanced mOLecular neTwork Analysis Supplementary Text

Alisa Pavel[1,2,3] Antonio Federico[1,2,3], Giusy del Giudice[1,2,3] Angela Serra[1,2,3], Dario Greco[1,2,3,4,*]

[1]*Faculty of Medicine and Health Technology, Tampere University, Tampere, Finland*

[2]*BioMediTech Institute, Tampere University, Tampere, Finland*

[3]*Finnish Hub for Development and Validation of Integrated Approaches (FHAIVE), Faculty of Medicine and Health Technology, Tampere University, Finland*

[4]*Institute of Biotechnology, University of Helsinki, Helsinki, Finland*

* *Correspondence: dario.greco@tuni.fi;*

Index

Index

Implementation
- Network Similarity and Distance Module
- Network Clustering Module
- Community Module
- Identification of Common Sub-patterns Module
- Network Simplification Module
- Plotting Module
- Pipeline Wrappers Module and Analysis Pipelines

Comparison to other Tools

Performance

Application / Case Study
- Network Inference
- Comparison of two networks
- Network Clustering
- Investigating a group of networks

1. Implementation

VOLTA is a Python package, containing 158 exposed functions, implemented in 7 modules (Figures 1 & 2) that can be used for different network-based analysis studies. While VOLTA can be applied to any type of (undirected) networks (following the given formatting requirements), it is focused on the application in multi-network comparison. VOLTA is implemented in Python 3.6 +, supports Linux, Mac and Windows systems and is published under a GNU GENERAL PUBLIC LICENSE Version 3 licence (individual parts may be published under different licenses, which can be inferred from the below mentioned packages).
VOLTA integrates many existing network libraries, which often only focus on a single task, into a combined library while providing additional functions targeted at differential network analysis. Following Python packages are used in VOLTA:

- NumPy >=1.17.* [1]
- Matplotlib >=3.0.* [2]
- statsmodels >= 0.11.* [3]
- Scikit-learn >=0.21.* [4]
- Brain Connectivity Toolbox for Python >= 0.5.* https://github.com/aestrivex/bctpy
- NetworkX >=2.5 [5]
- CDLIB >= 0.1.8 [6]
- SciPy >=1.3.* [7], [8]
- leidenalg >= 0.7 [9] https://github.com/vtraag/leidenalg
- seaborn >=0.9.* [10]
- pandas >=0.25 [11], [12]
- Markov Clustering >=0.0.6.* https://github.com/GuyAllard/markov_clustering [13]
- Cython https://cython.org/ [14]
- netneurotools >= 0.2.* https://github.com/netneurolab/netneurotools
- PyClustering == 0.9.3 [15]
- python-louvain >= 0.13 [16]
- treelib >= 1.6.* https://github.com/caesar0301/treelib
- pyintergraph https://gitlab.com/luerhard/pyintergraph
- Partition Quality == 0.0.7 https://github.com/GiulioRossetti/partition_quality
- ANGEL https://github.com/GiulioRossetti/ANGEL [17]

VOLTA has been tested on Ubuntu 18.04 with Python 3.6, Ubuntu 20.04 with Python 3.8, on Windows 10 with Python 3.7 and Python 3.8 and MAC OS 10.15.7 with Python 3.6.10.

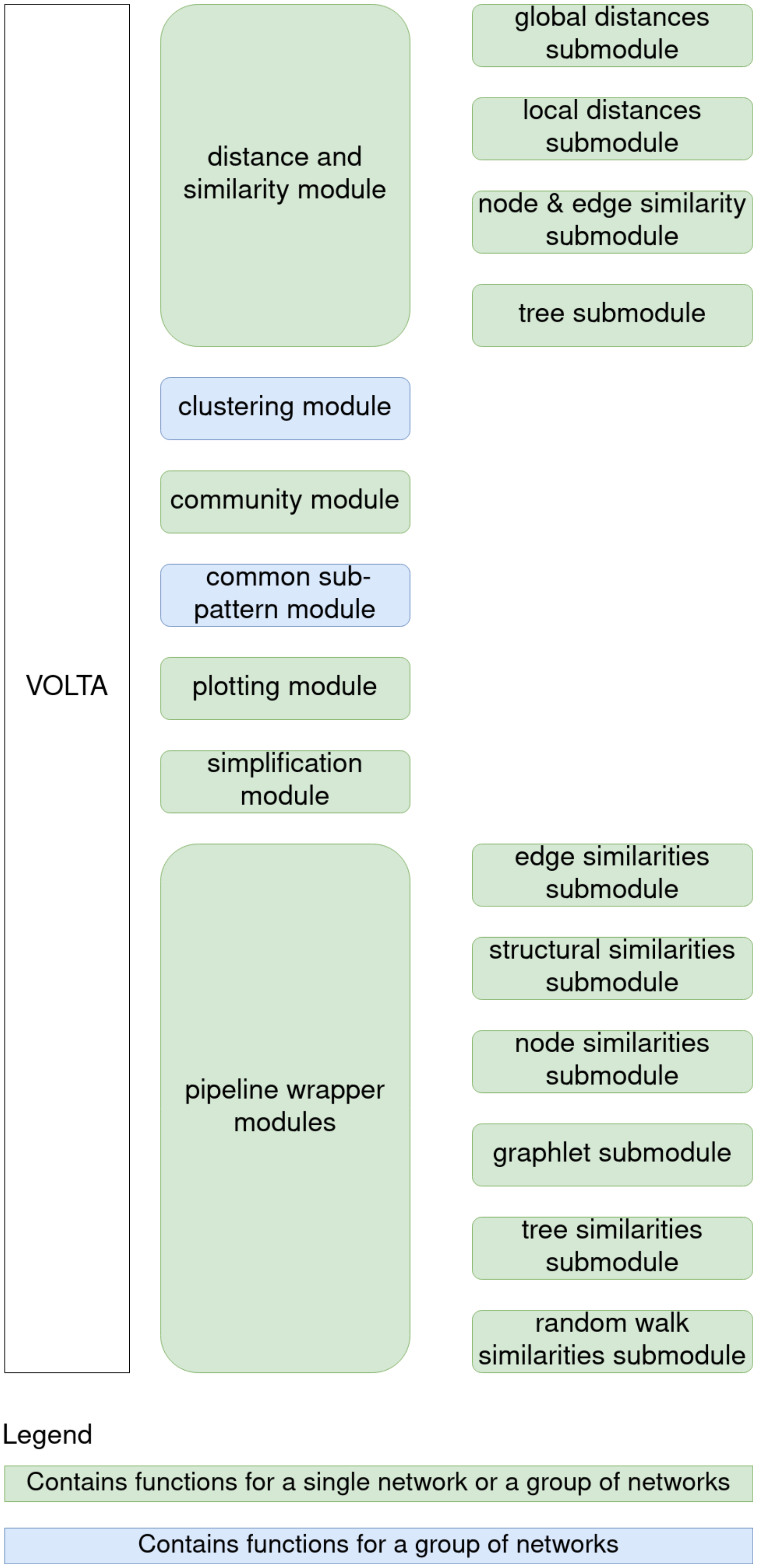

Figure 1: Module Structure of VOLTA

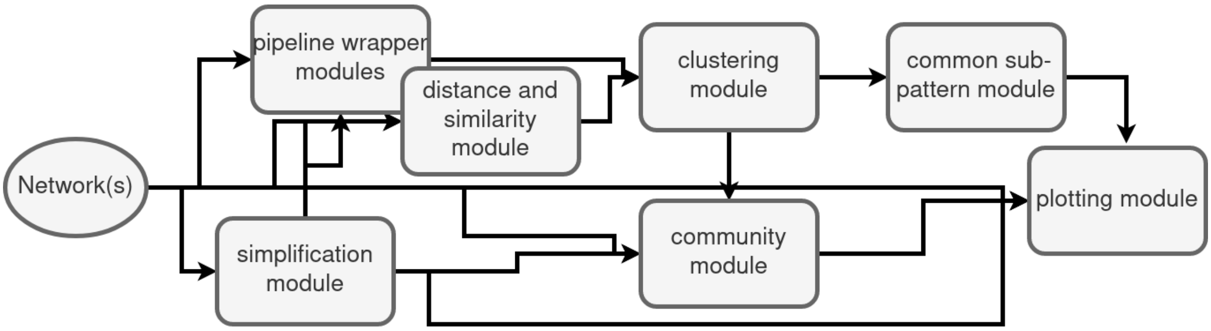

Figure 2: The modules of VOLTA and their possible pipeline interactions. The overlap of the pipeline wrapper modules and distance and similarity module indicates that most of the functions contained in the wrapper module call on functionalities from the distance and similarity module.

1.1. Network Similarity and Distance Module

Biological networks can provide insights into underlying processes in a biological system. For instance, they have been utilized to characterize the molecular basis of the onset of certain complex diseases, including cancer [18]–[21], type 2 diabetes [22]–[24], psoriasis [25], [26] and many others. Protein-protein interaction networks outline physical contacts within a system and analysing the network structure can give insight into associated functions and highlight regulatory proteins (genes) [27], [28]. In biological networks, the interplay between bio-molecules in a system is depicted. Comparing multiple such networks representing different biological conditions, can help to understand the complex underlying processes of diseases or depict the perturbations caused by the influence of exogenous compounds onto a biological system [29], [30].

Calculating distances between a group of networks allows to estimate which conditions are more similar and therefore may have similar effects onto the investigated system. Differences between networks can be calculated in a global or local manner. Global measures take the whole network into account, while local measures concentrate on sub-patterns. In VOLTA, different local and global measures are provided which can be investigated individually or in a combined manner. Since the different categories focus on different characteristics of a network, combining them provides deep insight into the similarities and differences between networks. A detailed example is provided in the case study (section 3).

The distance module contains 4 sub-modules (*global_distances, local, node_edge_similarities, trees*), which aim at estimating distances and / or similarities

between networks (or individual parameters that can be used to numerically describe a network [31]) as well as to estimate analysis parameters for single networks. It is a collection of network descriptive functions on a local, global and node-edge specific level.

The global distances submodule contains 17 exposed functions (https://github.com/fhaive/VOLTA/blob/master/html/volta/distances/global_distances.html), including functions to estimate a networks centrality distribution, clustering coefficient or metrics to classify a networks size. These functions are built on top of the NetworkX API [5]. Additionally, a similarity function based on random walks is provided, which compares node specific neighborhoods between networks based on their random walk patterns.

The local submodule contains 4 exposed functions (https://github.com/fhaive/VOLTA/blob/master/html/volta/distances/local.html), mainly classifying small subgraphs of the network known as motifs or graphlets [32], [33]. A node specific and node unspecific version is provided. The non node specific version allows to describe a networks topology based on its graphlet distribution, while the node specific version investigates if the same nodes are connected in the same way between networks.
Due to its complexity the node unspecific version can become computationally expensive on large networks. Therefore the function contains a parameter option to set an approximation threshold, which is the number of randomly selected nodes the graphlet distribution is estimated on. For each randomly selected node, node unspecific graphlets occurring in its neighborhood (node area) are counted. The neighborhood of a node is defined as all nodes connected by node size of the investigated graphlet steps from the randomly selected node. This method provides an estimation of small network pattern distribution over the network and can be used to describe a network's topology. The node specific version requires as input a list of nodes between which all possible graphlets of a given size are looked up in the investigated network. Graphlet based methods have previously found application in protein-protein interaction network description [28], [34] as well as in the classification of vascular networks [35].

The node and edge similarities submodule focuses on comparing two networks directly based on their nodes and edges and contains 24 exposed functions. Common and unique nodes or edges can be estimated between two networks on which different distance and/ or similarity measures, such as the jaccard index [36], Hamming distance [37] or SMC (Simple Matching Coefficient) [38], can be calculated. The users can make use of individual distances or a combination of distances. For example the jaccard index only considers shared nodes/ edges, while the SMC also considers shared non existing edges/ nodes. Depending on the performed analysis either only shared existing nodes/ edges may be of interest or also shared non-existing nodes/ edges. Therefore VOLTA allows the user to choose

based on their needs which (combination) of distance metrics to use, as well as to make a data driven decision based on a comparison of results of the different metrics.

Additionally, nodes and edges can be ranked by certain attributes, such as assigned weights or node/ edge centrality metrics, on which a correlation coefficient can be estimated (Kendall rank correlation coefficient [39]). Nodes can be ranked by their degree, closeness and betweenness centrality or a combination of these. For weighted networks edges can be ranked by either their assigned edge weights or edge weights can be estimated based on other metrics, such as their edge betweenness. Functions where asynchronous/ parallel computation can be applied are available to be run either in asynchronous or synchronous mode.

The tree module contains 8 exposed functions and provides functions to map a network structure to a binary tree. This can either be achieved by selecting a root node in the network or by hierarchically mapping loops contained in the network to a tree structure [40], [41]. Mapping a network structure to a tree, simplifies the computation problem and allows to investigate a network's connectivity with means of established binary tree metrics [35], such as its depth, asymmetry or branching ratio, which are provided in this sub-module. The functions in this sub-module are built upon the treelib (https://github.com/caesar0301/treelib) module to construct a tree object from the provided network.

The distances directly estimated between networks or calculated between their descriptive vectors, when transformed into a distance matrix (see https://github.com/fhaive/VOLTA/tree/master/jupyternotebooks), can be used as input for the clustering module (Figure 2).

1.2. Network Clustering Module

When a large group of networks is compared, making detailed network pairwise comparisons can be expensive. By pre-clustering the investigated networks into classes, instead the individual classes can be compared between each other or within each other. This is an extension of the use cases elaborated in 1.1 and an example is shown in section 3.

The clustering module contains 9 exposed functions, which can be used to cluster multiple networks based on distance/ similarity matrices as can be estimated from the functions described in 1.1. The module exposes hierarchical clustering and affinity propagation from scikit-learn [4]. The optics and k-medoids clustering algorithm are exposed from PyClustering [15]. Additionally a multiobjective function, whose objectives and parameters can be set by the user, is provided and can be used to tune the provided clustering algorithms as needed. To combine the results of different clustering algorithms or their individual runs a consensus clustering option is

implemented in a few variations which is built on netneurotools (https://github.com/netneurolab/netneurotools) and the underlying Brain Connectivity Toolbox (https://github.com/aestrivex/bctpy).

1.3. Community Module

Community detection algorithms aim at partitioning a graph into modules, based on its topology. This allows to, for example, identify functional groups in a co-expression network or protein-protein interaction network [29], [42], [43]. This module can be used to analyze a single network in search of gene modules, or to compare multiple networks based on their module similarities [44]. When comparing multiple networks, identified modules can be compared among them based on their network similarity (e.g. similarity of partitioning) or on a functional level (e.g. through functional enrichment) [29], [42], [45].

The community module contains 49 exposed functions, which cover community detection, consensus community detection and metrics to evaluate a graph partitioning. The implemented algorithms contain node clustering algorithms for weighted and unweighted networks as well as overlapping community detection algorithms. Many of the community detection algorithms are exposed from NetworkX [5], markov clustering (https://github.com/GuyAllard/markov_clustering), scikit-learn [4] and CDLIB [6] Python packages. The consensus algorithm, which allows to combine multiple different community detection algorithms or multiple runs of the same one, is based on the fast consensus clustering algorithm by Tandon et al. [46]. Different community detection algorithms detect communities based on different metrics, e.g. information flow in the network or modularity, which can yield highly different community structures based on the investigated network. A consensus strategy allows to combine the information from different algorithms that investigate the network from different biological point of views. Which community structure / detection method is the most suitable for the investigated networks and its analysis has to be decided by the user. As a guide VOLTAprovides multiple metrics to evaluate the network community partitioning ~~are implemented~~. Some of these metrics are built on functions implemented in the Partition Quality package (https://github.com/GiulioRossetti/partition_quality) as well as in NetworkX [5].

Community paritionings between multiple networks can be compared (based on their nodes, edges, etc.), with the functions implemented in the Network Similarity and Distance Module (1.1), by converting each individual community into a subgraph as shown in the example files.

In Table 1 there is a comparison of a subset of available community algorithms, some evaluation metrics, as well as showing the impact of a consensus strategy. *volta.communities.infomap()* [47] and *volta.communities.walktrap()* *[48]* are random walk based community detection algorithms. This group of algorithms detects

communities based on information flow within the network and tends to group nodes together which share many edges / information between each other. In the context of co-expression networks they group nodes together which have similar expression patterns. *volta.communities.label_propagation()* [49] assigns labels to nodes and propagates these through the network, and a nodes label is dependent on its neighbors labels. *volta.communities.louvain()* [50] identifies community structures based on an optimization of the modularity, which is a measure of the network density within a community vs. outside network density. *volta.communities.fast_consensus()* [46] is a consensus strategy based on the iterative agreement of selected algorithms/ community structures.

volta.communities.average_internal_degree() [51] estimates for each community its average internal node degree, a high internal degree indicates that the nodes within that community are strongly connected between each other. *volta.communities.conductance()* [52] measures for each community the fraction of edges leaving it, a small score indicates a strong conversion of information within a community. *volta.communities.fraction_of_weak_members()* [53] calculates the fraction of nodes within a community that have less inward edges than outward edges. A node with more outward than inward community edges indicates that this node is not a strong member of the community. *volta.communities.cut_ratio()* [54] measures for each community its cut ratio, which is the fraction of edges leaving the cluster out of all possible edges. *volta.communities.clustering_coefficient()* calculates for each community its clustering coefficient. A complete network has a clustering coefficient of 1. *volta.communities.get_number_of_communities()* returns the number of communities detected on a network and *volta.communities.get_number_of_nodes_communities()* returns the number of nodes contained within the communities as well as the mean community size.

Other community detection algorithms and evaluation metrics contained in VOLTA are outlined in the documentation (https://github.com/fhaive/VOLTA/blob/master/html/volta/communities.html).

Table 1: Four different community detection algorithms as well as a consensus (with *tresh=0.5*) are run on a random network of 100 nodes and 300 edges (created with NetworkX *gnm_random_graph(seed=12345)* [5]. The five communities are evaluated by means of five evaluation metrics. For each metric that calculates community specific scores the mean value of its communities is displayed. The different scores are displayed and either the highest or lowest score is marked in bold (depending on if the metric suggests low or high values). The consensus strategy returns the smallest amount of communities, containing on average the most nodes and achieves in three out of the five metrics the most desirable score.

	communities.inf	*communities.w*	*communities.la*	*communities.lo*	*communities.fa*

	omap()	*alktrap()*	*bel_propagation()*	*uvain()*	*st_consensus()*
communities.average_internal_degree()	2.67	2.6	2.63	3.05	**3.1**
communities.conductance()	0.54	0.54	0.55	0.49	**0.46**
communities.fraction_of_weak_members()	0.49	0.44	0.58	**0.357**	0.362
communities.cut_ratio()	0.59	0.59	0.61	0.56	**0.54**
communities.clustering_coefficient()	0.22	0.12	**0.38**	0.12	0.14
communities.get_number_of_communities()	10	9	8	7	6
communities.get_number_of_nodes_community()	10	11.11	12.5	14.29	16.67

1.4. Identification of Common Sub-patterns Module

When comparing a group of networks (e.g. co - expression networks under different conditions), common structures can be investigated, which could indicate functionalities that are not affected by the different exposures. This module can be used in combination with the clustering module, to identify (statistical) significant features that are present in a group but not in the others. Such an example is discussed in the case study in section 3.

This module contains 7 exposed functions, which aim to identify “common” subgraphs in a group of networks. Common subgraphs can either be identified on a set threshold, or statistically. The threshold method selects all edges that occur in at least a user-defined percent of the networks, to be considered as part of the common subgraph, and regards each edge as binary.

On the other hand, when applying the statistical method, a group of networks as well as a background distribution of networks needs to be provided. The background distribution informs on how many of all networks a specific edge exists, again regarding edges as binary. Each edge existing in a certain group of networks is evaluated against the background distribution in order to determine if it is statistically overrepresented in the selected group. Statistical significance is estimated based on a hypergeometric function and a Benjamini-Hochberg [55] correction is applied. Out

of the statistically overrepresented edges a common subgraph is constructed, which represents the statistical significant features of the selected group. Nodes that are not contained in at least one statistical overrepresented edge are not contained in the extracted subgraph.

The threshold based method works for any number of networks (> 1) that share at least one edge, while the statistical method requires a group of networks to be provided on which a background edge distribution can be calculated and a subset of all networks need to make up the investigated network group. This function can be used to characterize for example clusters of networks identified through the clustering module.

Additionally this module contains functions to estimate common community structures between a group of networks. Based on individual community paritionings of different networks a consensus partitioning can be created that represents the community partitioning of a group of networks, as implemented in the *get_consensus_community()* function. This function calls the *clustering.consensus_clustering()* function to estimate a consensus between the individual estimated network partitions. This function requires all networks to contain the same nodes.

1.5. Network Simplification Module

There can be different reasons why it can be helpful to simplify a given network. Network simplification means that a large (complex) network is reduced in its edges and/or nodes. For example, a path made of multiple nodes and edges can be reduced to a single edge between two nodes; edges with a low edge weight can be removed or a network can be reduced to its minimum spanning tree, where all nodes are connected with a minimum of edge (weights). Large networks may be computationally expensive and a simplification would lead to significant improvements. Further some topological features (especially when comparing multiple networks) may be visible only after network simplification (e.g. from a complete network to a more sparse network) and could allow to detect areas of interest between the networks. This reduction in complexity can also reduce computation time for other algorithms, such as the clustering module and additionally can help to identify differences and similarities between networks (topology) by only focusing on significant edges (depending on the simplification method significant edges are selected differently). Moreover, a reduced network is easier to visualize. For example, (topological) features of the network may be visualized better or only the most significant nodes/ edges can be displayed.

The network simplification module exposes 7 functions, which provide different functionalities to simplify a network through node or edge removal, adjust assigned edge weights or estimate a network's minimum spanning tree. For distance/ similarity

networks (networks where their assigned edge weights indicate either a distance or similarity) a function which converses between distances and similarities is provided and for correlation networks a function to change the edge weights to their absolute values is provided. Edges can either be removed globally or on a per node basis based on their edge attributes (either through a fixed threshold or by providing a percentage of edges to be removed) or through probabilistic sampling, which aims at identifying weak links between two nodes and removing them.

1.6. Plotting Module

The plotting module contains 7 exposed functions.
The clustering of networks (1.2) can be plotted on top of the provided distance matrix used for clustering, which provides a visual representation of cluster size distribution and within cluster similarity as well as between cluster dissimilarity (Figure 3A).
Additionally an agreement matrix between network clusterings (1.2) or community detection algorithms (1.3) can be plotted, which indicates for each network (or node) pair in how many of the performed algorithms they are grouped together (Figure 3B). Different network partitionings (1.3) or clusterings (1.2) can be compared by means of correlation and plotted into a hierarchical cluster map (Figure 3C). The investigated networks and communities can be visualized (Figure 3D and 3E), which is built on top of the NetworkX [5] plotting functions. Other plotting functions such as heatmaps are also provided. These functions are built up on the seaborns [10] and matplotlib [2] API.

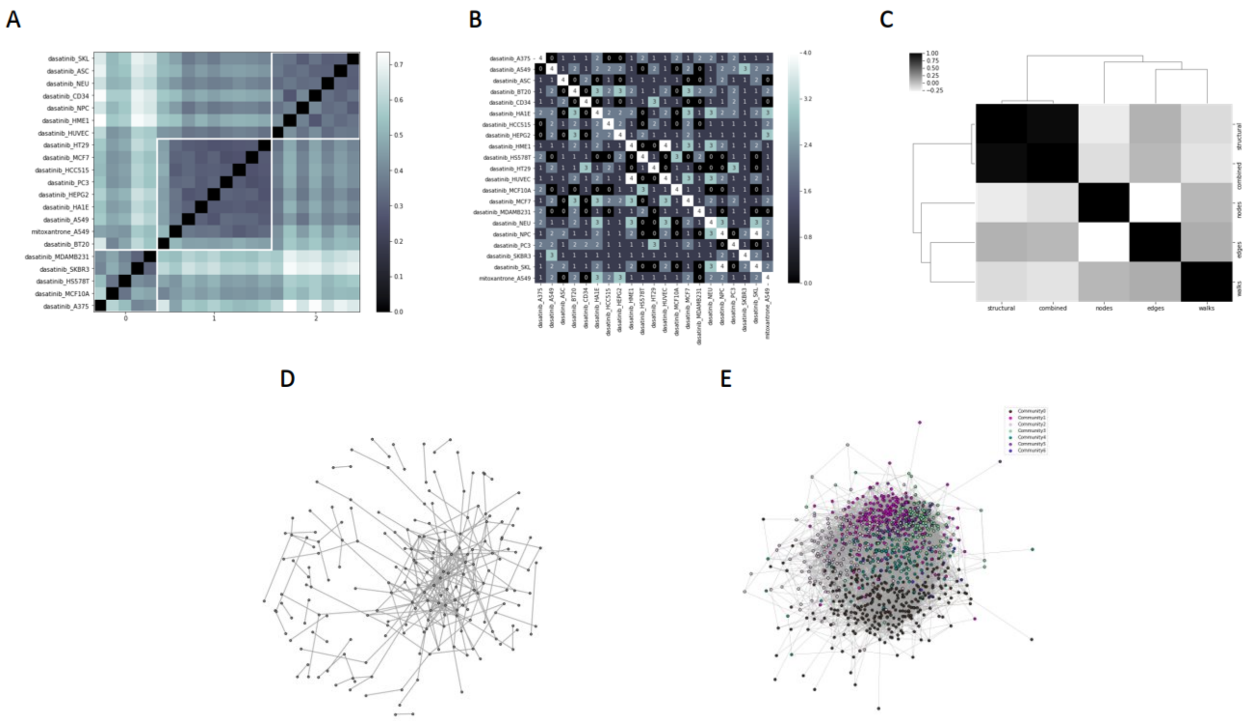

Figure 3: Example of plots that can be generated with VOLTA. A) A clustering is plotted on top of a provided distance matrix. In this example the 21 networks are grouped into 3 clusters (white squares on the matrix diagonal) B) An agreement matrix between 4 different network clusterings algorithms can be plotted. In this example, 4 indicates agreement between all the algorithms, while 1 indicates that only 1 of the algorithms has grouped two networks together. This plot provides the user with an insight in how robust the different biological views are for each network pair. A high agreement indicates that this specific network pair is similar across most of the investigated biological dimensions while a low agreement indicates that they are different in most of the investigated dimensions. The user can decide based on this insight if they want to investigate some dimensions in more detail for specific data sets as well as to get an insight in the agreement between the different investigated biological dimensions. C) The Pearson correlation between different clusterings or network partitionings can be plotted. In this example 5 different clusterings are compared. This plot quantifies how different the individual network views are from each other. A high correlation indicates that the networks are highly similar independent from the investigated biological angel. Additionally it allows the users to identify how similar the combined view is to the individual views and based on this decide if a single (or subset) of dimensions would provide the same result/ have the most impact. If the individual dimensions are highly different the user can decide to investigate them individually (or a subset) instead. D) The investigated network can be plotted. E) Identified communities can be plotted on top of the network.

1.7. Pipeline Wrappers Module and Analysis Pipelines

The pipeline wrappers module contains 6 submodules, which aim at providing wrapper functions for the different analysis steps (for multiple networks) that can be performed during a co-expression network analysis. The purpose of providing wrapper functions and whole analysis pipeline files is to make the package easy to use for novice users, being it computationally or in co-expression network analysis. This allows users to replicate analyses easily between studies and allows new users to run a complete analysis pipeline with a minimal amount of direct interactions with VOLTA. While on the other hand exposing the individual functions in a Python package allows more experienced users to run their own analysis, to adjust parameters in more detail as well as to use VOLTA for other network analysis tasks than on co-expression networks.

Three analysis pipelines are defined, in addition to a *community detection*, *functional enrichment* and a *read-and-write graph format file*.

1.7.1. Submodules

The six submodules contain wrapper functions for estimating distance/ similarity matrices between multiple networks based on their nodes, edges and structural properties as well as based on random walks and the implemented tree functions (1.1). The wrappers allow to call multiple metrics through an individual function call, as well as to compute the same measurements for multiple networks simultaneously and to translate the results into distance/ similarity matrices where applicable. In total there are 22 exposed functions distributed over six submodules.The individual functions are implemented in the distance module (1.1). The application of these submodules is shown in the analysis pipeline files.

1.7.2. Analysis Pipelines

The VOLTA package provides 3 complete examples of analysis pipelines in the form of Jupyter Notebooks (https://github.com/fhaive/VOLTA/tree/master/jupyternotebooks). Moreover, examples are provided to: (i) show how to read and write different graph formats to be processed by VOLTA [https://github.com/fhaive/VOLTA/blob/master/jupyternotebooks/import_and_export_of_networks.ipynb]; (ii) show how to use the community module (1.3) [https://github.com/fhaive/VOLTA/blob/master/jupyternotebooks/community_detection.ipynb]; (iii) show how to functionally enrich a set of genes through the Panther enrichment API (http://pantherdb.org/) [56], [57] [https://github.com/fhaive/VOLTA/blob/master/jupyternotebooks/Example_of_Enrichment.ipynb]. The provided files are structured in such a way that users will only need to exchange the example networks with their own networks in order to perform a default analysis. By providing the pipelines as Jupyter Notebook files it is very easy for inexperienced users to modify small parts of the pipeline, such as changing

parameters, adding or removing individual steps or even adding external analysis packages, while being provided with visual output and an interactive way to interact with VOLTA. Additionally, we added explanations on how to interpret the results of each step as well as what further steps could be performed or how the analysis could be expanded to each function, used in the provided default analysis pipelines.

The 3 analysis pipelines defined, can be run individually or part of an extended analysis. First a **network - network** comparison analysis pipeline is provided (https://github.com/fhaive/VOLTA/blob/master/jupyternotebooks/network_-_network_comparison.ipynb), which compares two or more networks based on their node and edge properties, their individual node sub-areas and communities. A file that shows how to use the community module (1.3) is provided (https://github.com/fhaive/VOLTA/blob/master/jupyternotebooks/community_detection.ipynb), which if desired by the user, can replace the simple community detection section in the network - network comparison analysis pipeline. This pipeline makes use of the *get_node_similarity*, *node_edge_similarities*, *get_edge_similarities* and *get_walk_distances* pipeline wrapper submodules.
Secondly, a **network clustering** pipeline is provided (https://github.com/fhaive/VOLTA/blob/master/jupyternotebooks/Network_clustering.ipynb), which shows how multiple networks can be grouped based on their nodes, edges, subgraph and structural properties. This pipeline mainly makes use of the functions defined in the distance module (1.1) as well as the clustering module (1.2). The groupings resulting from this pipeline can be given as input for the third defined analysis or a separate group of networks can be provided. This pipeline file calls functions contained in the *get_node_similarity*, *get_edge_similarities, get_structural_vector* and *get_walk_distances* pipeline wrapper submodules.
This analysis aims at identifying **common subgraphs** in a group of networks as well as a consensus community partitioning of a group of networks and mainly makes use of functions implemented in the common sub pattern module (1.4) (https://github.com/fhaive/VOLTA/blob/master/jupyternotebooks/Are_there_common_strucutures_or_communities_in_a_group_of_networks_.ipynb).
The network simplification module (1.5) can be integrated with any of these analyses in order to pre-modify the networks before performing elaborate analysis (Figure 2).

2. Comparison to other Tools

~~To date, many network analysis software solutions and packages have been published. Due to the versatile nature of network models, either very general packages that focus on basic network computations and metrics (such as NetworkX [5] and iGraph [58]) or specialized packages for a particular problem (such as CDLIB [6], which focuses on community detection) have been published. Software solutions for co-expression network analysis often are optimized for a single analysis pipeline or step and are commonly provided as a whole software solution (such as INfORM [45] or CoNekt [59]). These softwares are usually easy to use and to understand for~~

novice users. However, they do not provide users with the flexibility to use the software for other than the intended analysis (resulting from non exposed functions, non modifiable pipelines and fixed file formats).

We therefore developed VOLTA an easy to use and flexible package that combines the strengths of these software groups. VOLTA combines a diverse set of functions, regarding many different fields of network analysis. Moreover, VOLTA aims, when possible, to provide different algorithms for a given task (e.g. community detection algorithms). This allows users to customize their pipelines, for example based on their network structure or aims. Moreover, the user can apply ensemble strategies which have been proven to give more robust results [45], [46]. For example in community detection the results depend on the algorithm as well as the structure of the network, since different algorithms might use different metrics to select a partitioning. Similarly, in clustering, different algorithms can yield different results when applied to the same data. VOLTA provides metrics (https://github.com/fhaive/VOLTA/tree/master/html/volta) to evaluate these algorithms, allowing users to select the best methods for their data set(s). All these functions are fully exposed in the package, allowing users to set their own parameters and combine them as needed. Additionally, pre-defined pipelines for specific analysis in the domain of co-expression networks are provided. These are provided in Jupyter Notebook files, which make it easy for the user to modify them and at the same time to have a complete report of the analysis in the same file. This allows inexperienced users to plug and play with VOLTA, while experienced users have the possibility to construct their own unique analysis pipelines.

Table 2 and Table 3 compare VOLTA with different common network packages and co-expression network analysis software in terms of their included functionalities, flexibility and user-friendliness. The most similar tool to VOLTA is CompNet [60], which is a Perl/ R based software application for the purpose of visual network comparison available for Windows and Linux systems, mainly running on the R iGraph library. Since CompNet is a software application its functionalities are non exposed and cannot be used in a different manner than provided in its interface, input formats are restricted and availability across systems is limited. This implies that it is difficult to integrate its functionalities into larger analysis pipelines or to use it in combination with other applications.

Due to this VOLTA is a unique application, which can be used by a wide range of user groups for a diverse set of (co-expression) network analysis without the need of combining numerous available software solutions and to worry about their compatibility. VOLTA's implementation allows it to be applied to any type of network that can be represented as a NetworkX Graph object; this can be undirected, directed, unipartite or bipartite networks. However the main target of VOLTA are undirected, unipartite co-expression networks, which indicates that for other types of networks the user needs to decide which functions and in which combination to

apply to their networks. A subset of the provided functions can be applied to directed networks, which is indicated in their documentation. So for example VOLTA can be used to analyse social networks, regulation networks, genetic interaction networks [61], [62], the clustering of protein-protein interaction networks [63] or to analyze the role of metabolites in a system [64].

Table 2: Comparison to Other Network Packages based on individual functionalities

Tool	Language	Community Functions	Network Metrics	Network Similarity Estimation & Clustering	Identification of Common Sub-structures	Network Simplification
NetworkX [5]	Python	some	**yes**	no	no	**yes**
iGraph [58]	Python/ R	**yes**	**yes**	no	no	**yes**
WGCNA [65]	R	**yes**	**yes**	no	some	N/A
CDLIB [6]	Python	**yes**	no	no	no	no
BioNetStat [66]	R	no	some	some	no	some
INfORM [45]	R	some	some	no	no	some
CoNekT [59]	Python / JavaScript	some	some	some	some	N/A
CompNet [60]	Perl/ R	some	some	**yes**	**yes**	N/A
NetSimile [31]	N/A (no official implementation)	no	**yes**	**yes**	no	no
VOLTA	Python	**yes**	**yes**	**yes**	**yes**	**yes**

Table 3: Comparison to Other Network Packages based suitability for inexperienced and experienced users as well as versatility

Tool	Provides Predefined Analysis Pipelines?	Exposing Individual Functions & their Parameters?	Package Usage Examples Provided?	Suited for Co-expression Network Analysis?	Suited for Different Types of Networks?	File Format Restrictions?
NetworkX [5]	no	**yes**	**yes**	some functionalities	**yes**	**no**
iGraph [58]	no	**yes**	**yes**	some	**yes**	**no**

				functionalities		
WGCNA [65]	some	**yes**	**yes**	**yes**	no	yes
CDLIB [6]	no	**yes**	**yes**	some functionalities	**yes**	**no** (as in NetworX/ iGraph)
BioNetStat [66]	**yes**	no	**yes**	**yes**	no	yes
INfORM [45]	**yes**	no	**yes**	**yes**	no	yes
CoNekT [59]	**yes**	no	**yes**	**yes**	no	yes
CompNet [60]	N/A	no (software application)	**yes**	some functionalities (network comparison only)	**yes**	yes
NetSimile [31]	N/A (no official implementation)	N/A (no official implementation)	N/A (no official implementation)	some functionalities	**yes**	N/A (no official implementation)
VOLTA	**yes**	**yes**	**yes**	**yes**	**yes**	**no** (as in NetworkX)

3. Performance

To evaluate the impact of variation in network size and number of networks on computation time as well as memory usage, performance profiling is performed on the network clustering (https://github.com/fhaive/VOLTA/blob/master/jupyternotebooks/Network_clustering.ipynb) (Figures 4-7), network comparison (https://github.com/fhaive/VOLTA/blob/master/jupyternotebooks/network_-_network_comparison.ipynb) (Figures 8-10), community detection (https://github.com/fhaive/VOLTA/blob/master/jupyternotebooks/community_detection.ipynb) (Figures 11-13) and common substructures (https://github.com/fhaive/VOLTA/blob/master/jupyternotebooks/Are_there_common_strucutures_or_communities_in_a_group_of_networks_.ipynb) (Figures 14-16) pipeline. Synthetic gene expression values have been created with MVBioDataSim [67]. From these expression values pairwise Pearson correlations have been calculated and the top ranked edges, based on absolute correlation values, have been selected in order to create networks of a desired density. If not stated otherwise the selected edges are considered to be unweighted and undirected.

Computation time of the complete pipelines are measured as well as the memory usage over time. Memory usage is measured with the Python memory-profiler

package through its mprof method, which measures the allocated memory of the script every 0.1 seconds.

Clustering Pipeline

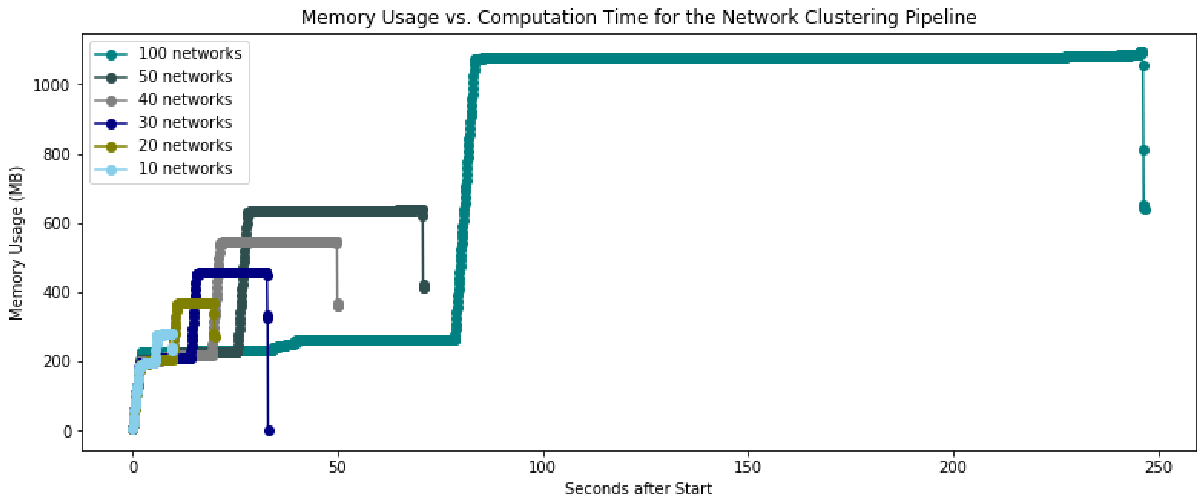

Figure 4: Allocated memory at each time point for the Network clustering pipeline for a group of 10, 20, 30, 40, 50 and 100 networks. Each network contains 100 nodes and a density of 0.2.

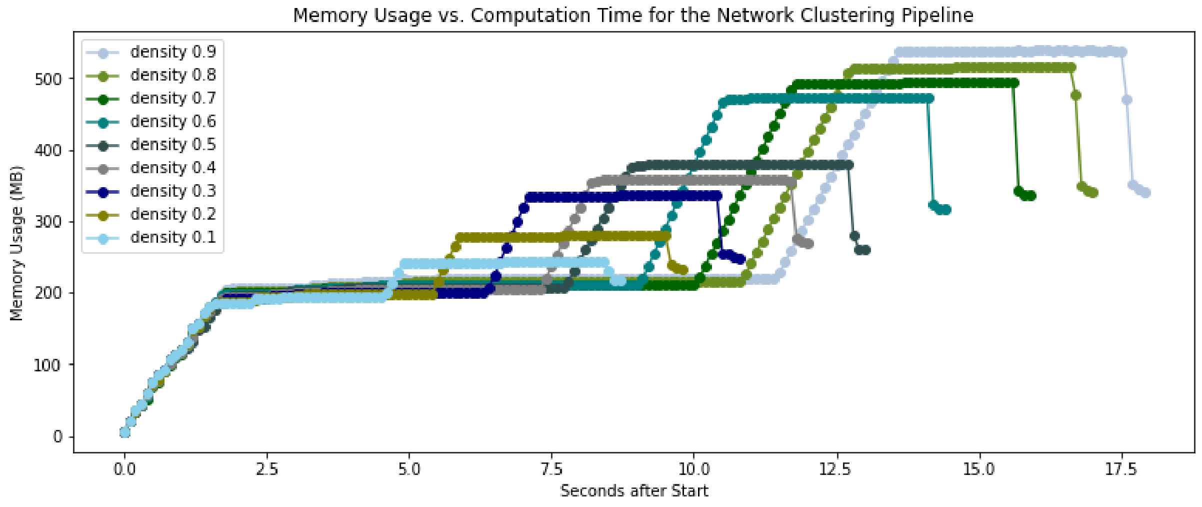

Figure 5: Allocated memory at each time point for the network clustering pipeline for groups of 10 networks of 100 nodes with varying densities between 0.1 to 0.9.

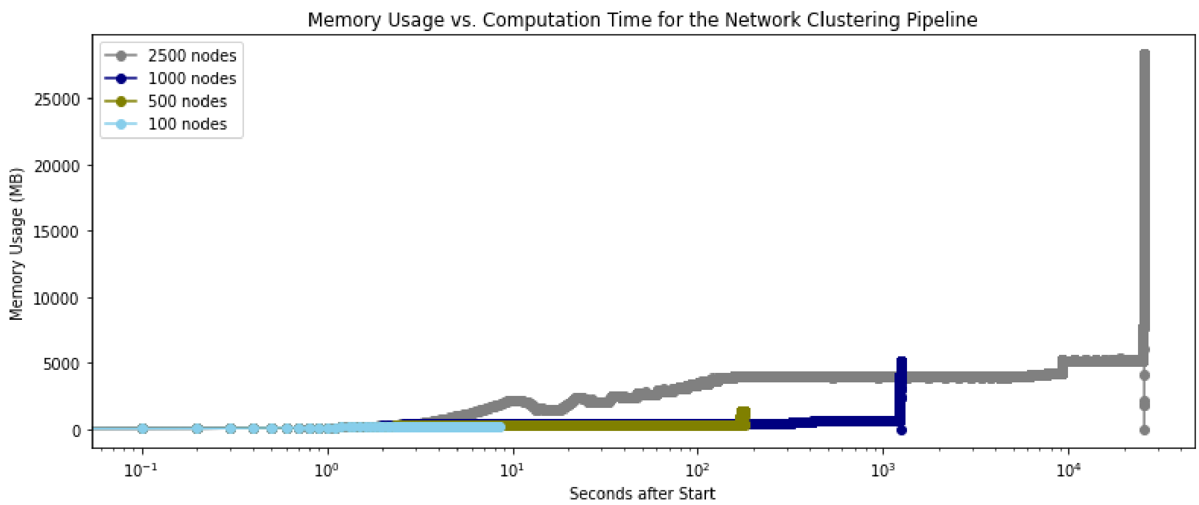

Figure 6: Allocated memory at each time point for the network clustering pipeline for groups of 10 networks with density 0.1 and varying node sizes of 100, 500 1000 and 2500 nodes.

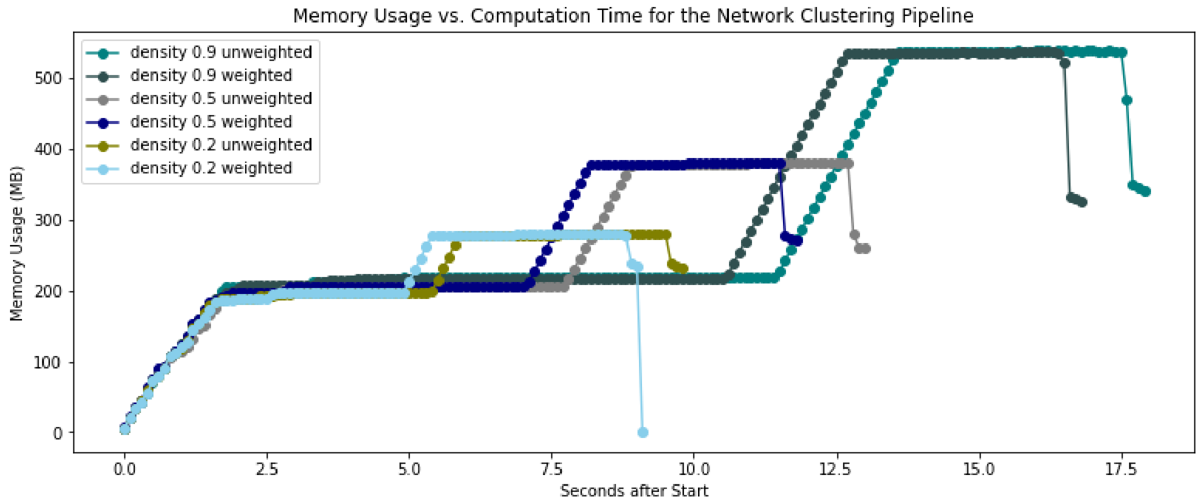

Figure 7: Allocated memory at each time point for the network clustering pipeline for groups of 10 networks of unweighted and weighted networks of density 0.2, 0.5 and 0.9 with 100 nodes each. For the weighted networks the absolute Pearson correlation values p and 1-p are assigned and used depending on if the used algorithm expects a distance or similarity value.

Network Comparison Pipeline

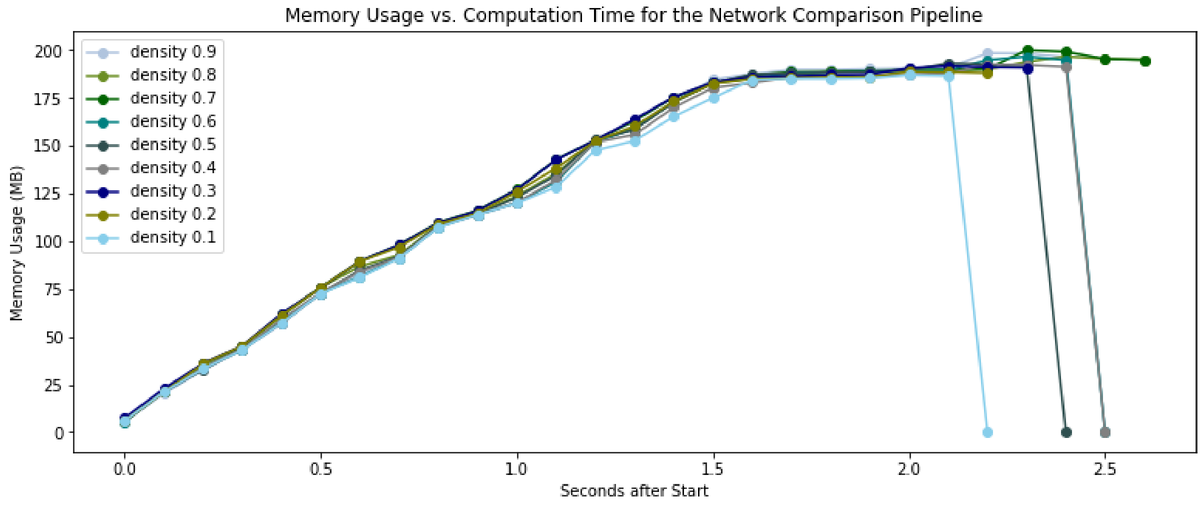

Figure 8: Allocated memory at each time point for the network comparison pipeline for 2 networks of 100 nodes with varying densities between 0.1 to 0.9.

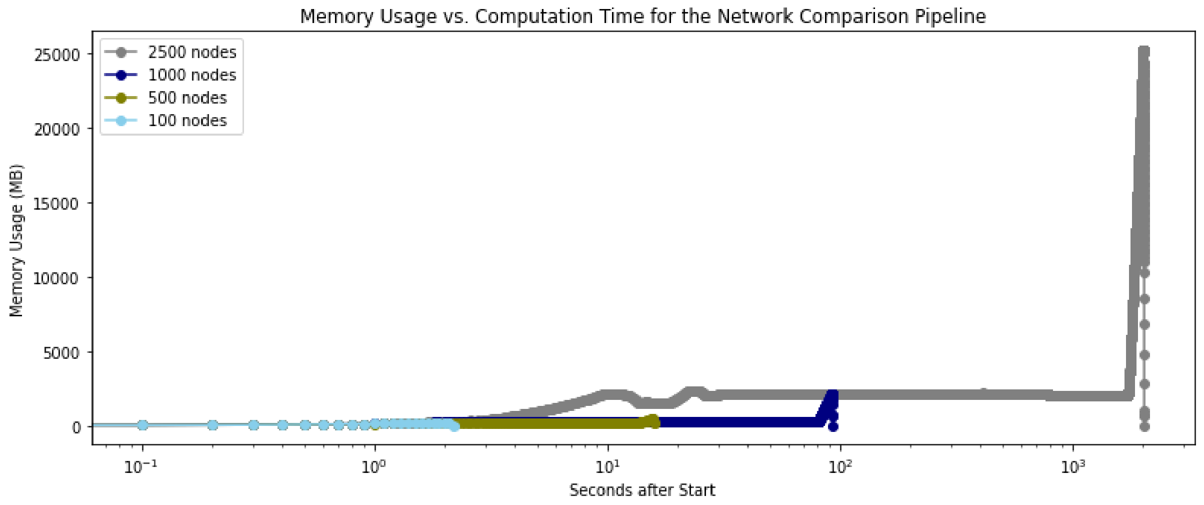

Figure 9: Allocated memory at each time point for the network comparison pipeline for 2 networks with density 0.1 and varying node sizes of 100, 500, 1000 and 2500 nodes.

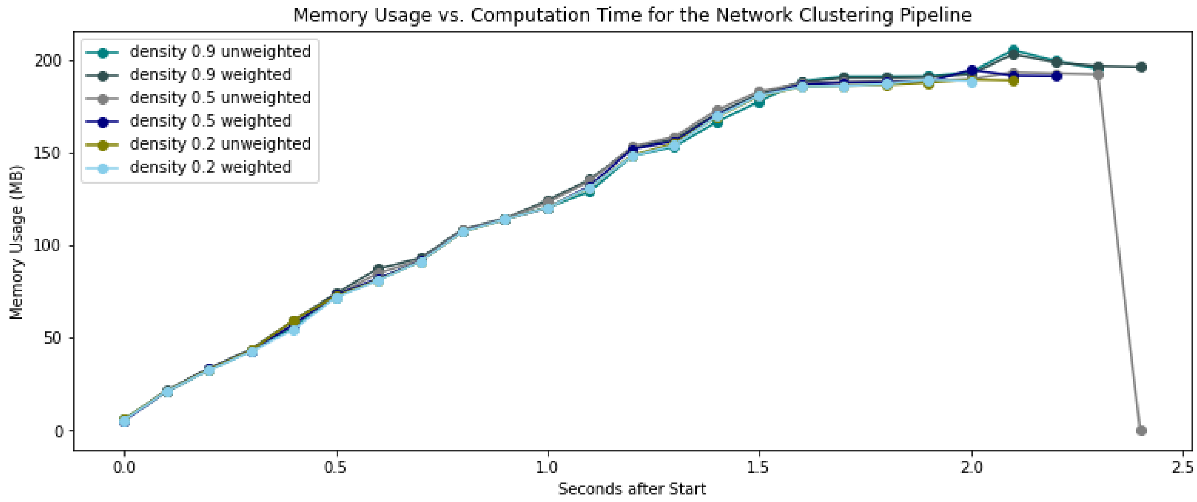

Figure 10: Allocated memory at each time point for the network comparison pipeline for groups of 10 networks of unweighted and weighted networks of density 0.2, 0.5 and 0.9 with 100 nodes each. For the weighted networks the absolute Pearson correlation values p and 1-p are assigned and used depending on if the used algorithm expects a distance or similarity value.

Community Detection Pipeline

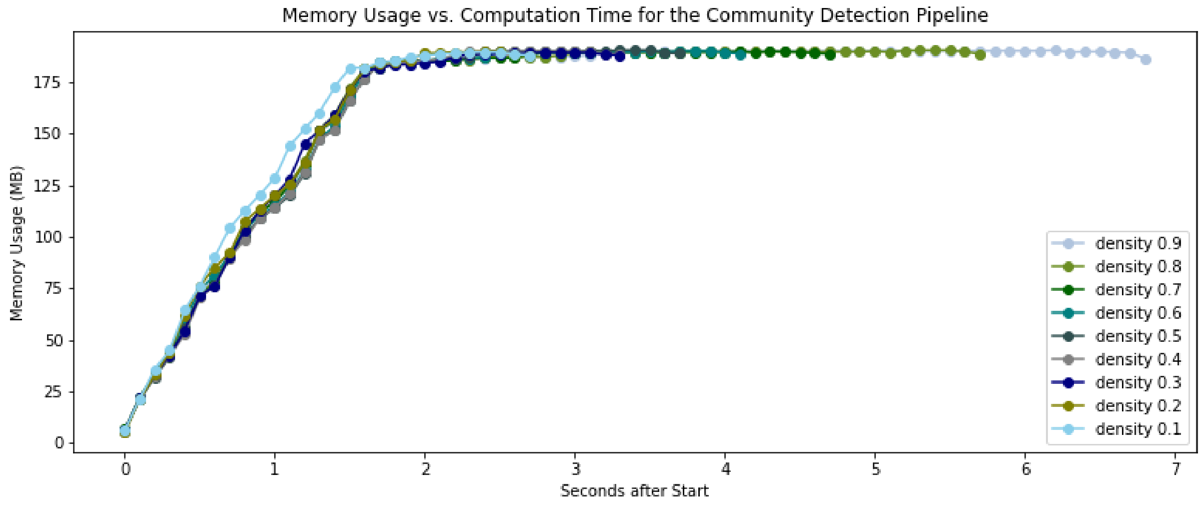

Figure 11: Allocated memory at each time point for the community detection pipeline for 1 network of 100 nodes with varying densities between 0.1 to 0.9.

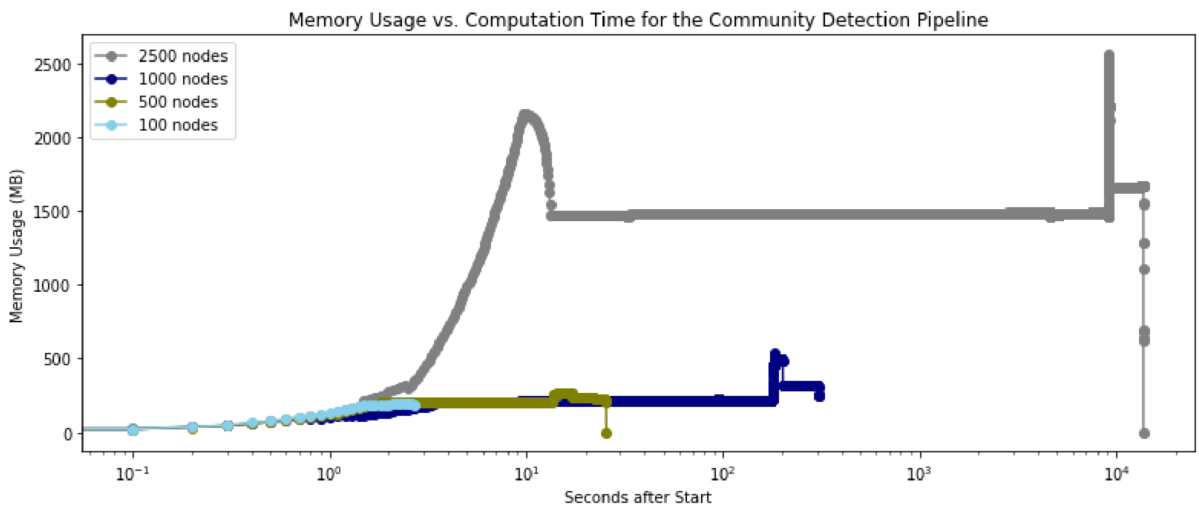

Figure 12: Allocated memory at each time point for the community detection pipeline for 2 networks with density 0.1 and varying node sizes of 100, 500, 1000 and 2500 nodes.

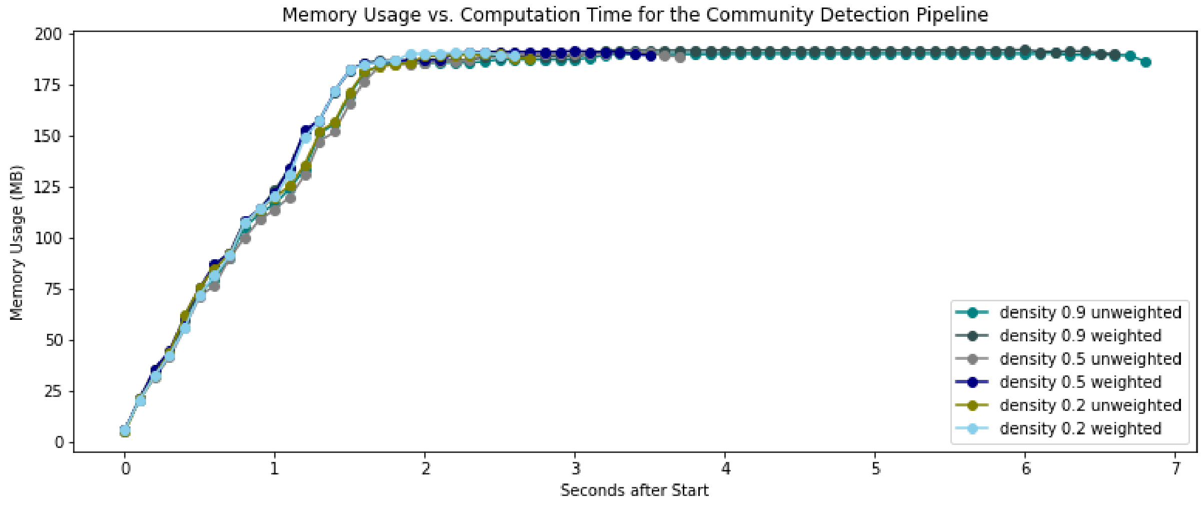

Figure 13: Allocated memory at each time point for the community detection pipeline for groups of 10 networks of unweighted and weighted networks of density 0.2, 0.5 and 0.9 with 100 nodes each. For the weighted networks the absolute Pearson correlation values p and 1-p are assigned and used depending on if the used algorithm expects a distance or similarity value.

Common Subgraphs Pipeline

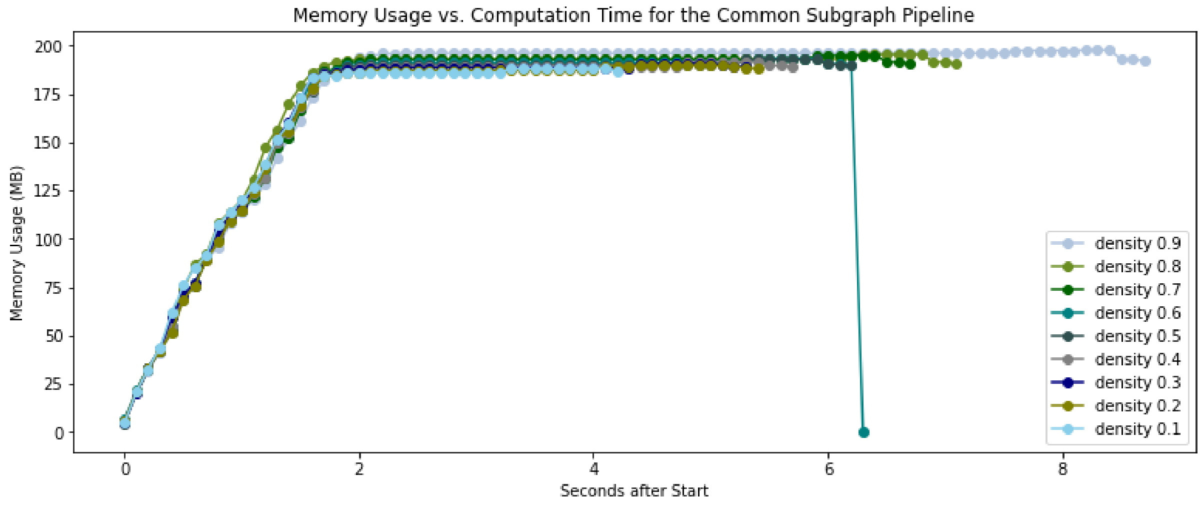

Figure 14: Allocated memory at each time point for the common subgraphs pipeline for 5 networks of 100 nodes with varying densities between 0.1 to 0.9.

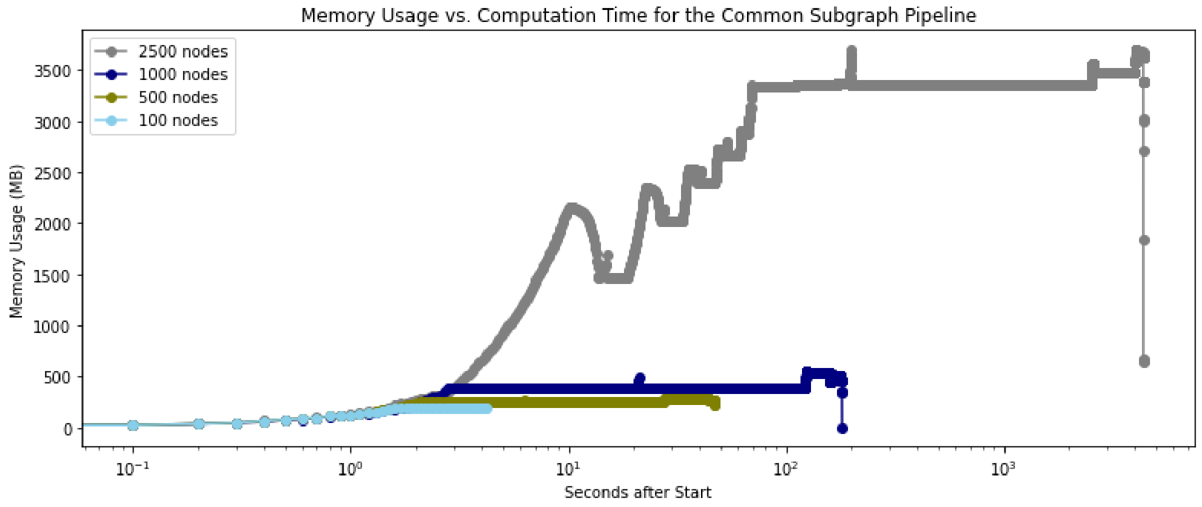

Figure 15: Allocated memory at each time point for the common subgraphs pipeline for 5 networks with density 0.1 and varying node sizes of 100, 500, 1000 and 2500 nodes.

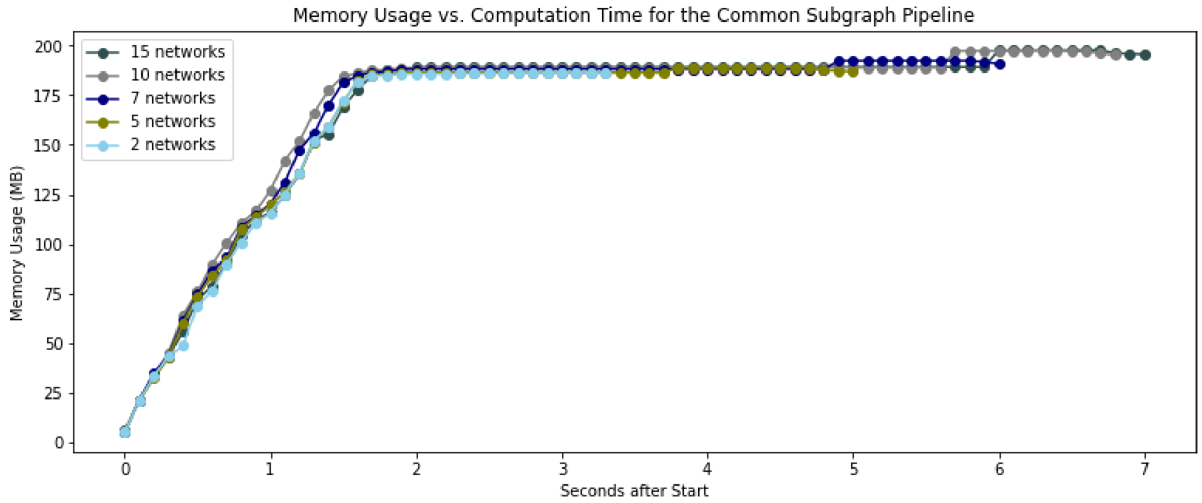

Figure 16: Allocated memory at each time point for the common subgraphs pipeline for a group of 2, 5, 7, 10 and 15 networks. Each network contains 100 nodes and a density of 0.2.

For all the pipelines, the computation time increases with an increase in network size or number of networks. The same applies to the increase in memory usage for the network clustering and network comparison pipeline, while network density does not yield a significant increase in memory allocation for the community detection and common subgraph pipelines. Weighted networks require reduce computation time, which likely mainly results from the fact that the network edges are compared based on their edge values instead of edge betweenness scores, which would need to be computed within the pipeline. For the common subgraph pipeline, no weight based profiling is performed, since its individual algorithms are weight independent. The pipelines are run as presented in the jupyter files, with all network objects loaded into memory (instead of making use of the reading from file option) and the asynchronous option set to false where applicable.

4. Application / Case Study

We showcase the main functionalities of the VOLTA package through two different examples of analyses. In the first example, we showcase the functionality of the VOLTA package for the comparison of two co-expression networks. We used two networks representing A549 cells treated with dasatinib and mitoxantrone, a Tyrosine kinase inhibitor and a cytostatic drug, respectively. With this analysis we aim to highlight the differences in the mechanism of action of the two drugs acting on the same cell line. In the second example, we showcase the functionality of the VOLTA package for grouping multiple co-expression networks. We collected co-expression networks of 20 cell lines after treatment with dasatinib (Table 4). By analyzing these networks, we aim to characterize the responses of different cell lines to the same drug treatment.

The different networks and their respective dimensionalities are displayed in Table 4.

Table 4: The networks used in the case study and their network size.

Cell Line	Treated with	# Nodes	# Edges	Density
HEPG2	dasatinib	977	11032	0.023
HCC515	dasatinib	977	13543	0.028
MCF7	dasatinib	977	13102	0.027
PC3	dasatinib	977	12775	0.026
HA1E	dasatinib	977	12405	0.026
HT29	dasatinib	977	11588	0.024
A549	dasatinib	977	10820	0.022
A549	mitoxantrone	977	15308	0.032
MCF10A	dasatinib	977	13942	0.029
BT20	dasatinib	977	10605	0.022
HS578T	dasatinib	977	17535	0.036
A375	dasatinib	977	27178	0.057
SKBR3	dasatinib	977	28388	0.059
MDAMB231	dasatinib	977	25135	0.052
NPC	dasatinib	977	7857	0.016
NEU	dasatinib	977	6994	0.014
SKL	dasatinib	977	6710	0.014
CD34	dasatinib	977	4294	0.009
ASC	dasatinib	977	5772	0.012
HUVEC	dasatinib	977	9828	0.020
HME1	dasatinib	977	4093	0.008

4.1. Network Inference

In order to infer the networks utilized in this case study we retrieved the L1000 Connectivity Map perturbational profiles from the Gene Expression Omnibus (GEO) (GEO ID: GSE70138). The dose and time point of drug perturbation for the present case study are 10 um and 24h. In order to infer the networks, only the transcriptional signatures of the L1000 landmark genes were employed (for more information, please read [68]). Such signatures were utilized as input to INfORM [45], a comprehensive tool for the inference of robust co-expression networks through an ensemble approach. The algorithms selected in INfORM to infer the networks are clr [69], ARACNE [70] and MRNET [71], while the metrics of correlation to build the adjacency matrices are Pearson correlation, Spearman correlation, Kendall correlation, empirical mutual information, Miller-Madow asymptotic bias corrected empirical estimator, Schurmann-Grassberger estimate of the entropy of a Dirichlet probability distribution and a shrinkage estimate of the entropy of a Dirichlet probability distribution, as implemented in the R minet package [72]. The resulting networks are undirected, non-complete binary networks.
The co-expression networks were converted into edgelists compatible with the NetworkX graph format, as shown in the import and export Jupyter Notebook (https://github.com/fhaive/VOLTA/tree/master/jupyternotebooks).

4.2. Comparison of two networks

To showcase the capabilities of the VOLTA package to compare two networks, we analyzed the co-expression networks built from the A549 cell line treated with dasatinib and mitoxantrone. This comparative analysis allows us to evaluate the changes taking place in the networks induced by the two treatments. As a first characterization, we sought to find genes whose centrality is remarkably different in the two networks. To do so, we calculated the centrality of each node of the two networks by computing the median among several centrality metrics, including degree centrality, betweenness centrality and closeness centrality via the *distances.node_edge_similarities.sort_node_list()* function. We, then, ranked the nodes on the basis of the median centrality value and estimate the rank differences for each gene between the two networks.

As a result, we observed that the *OXA1L*, *YME1L1* and *DNAJC15* genes have a higher centrality in the mitoxantrone network compared with the dasatinib network where they have a less central role. These results suggest an involvement of mitoxantrone in the impairment of mitochondrial proteins metabolism, as also previously demonstrated in [73]. On the contrary, the genes *KEAP1* and *NVL* show the opposite trend, acting as top central genes in the dasatinib network and resulting peripheric in the mitoxantrone one. While *KEAP1* gene is associated with Papillary carcinoma and Goiter, Multinodular 1, with or Without Sertoli-Leydig Cell Tumors, the

gene *NVL* has never been associated with cancer or with dasatinib mechanism of action. The values of the median centrality in both of the networks, along with the absolute centrality difference of the top 10 differentially central genes are reported in Table 5.

Table 5: Top 10 nodes ranked in their differences of median centrality rankings in the individual networks.

Entrez ID	Gene Symbol	Absolute Ranking Difference	Dasatinib Ranking	Mitoxantrone Ranking
5018	*OXA1L*	924	964	40
10730	*YME1L1*	921	946	25
9817	*KEAP1*	895	19	914
4931	*NVL*	884	60	944
29103	*DNAJC15*	876	927	51
54555	*DDX49*	863	43	906
51097	*SCCPDH*	858	96	954
1070	*CETN3*	851	12	863
26136	*TES*	839	128	967
29978	*UBQLN2*	838	83	921

In order to obtain a biological insight behind these connectivity pattern changes in both of the networks, we interrogated the WEB-based Gene SeT AnaLysis Toolkit (WebGestalt [74] http://www.webgestalt.org/) by performing a Gene Set Enrichment Analysis (GSEA) on the complete ranked gene list, of which the top genes are reported in Table 5 against the Reactome database [75] (run on Dec. 8th 2020). The results showed that the biological processes that are sustained by genes whose centrality is significantly affected by the drug treatments are mostly related with cell cycle and mitotic processes as well as signaling cascades led by the MAPK and Tyrosine kinase activities, which are in line with the core activities of both of the drugs.

Next we investigate for each node, if changes in their neighborhood have occured. For each common node between the two networks 10 times degree random walks of length 5 are performed via the *example_pipeline_wrappers.get_walk_distances.helper_walks()* function. Estimating

for each node the number of walks performed based on its degree ensures that the results are comparable between nodes. The more walks are performed, the more accurate the subgraph area is explored. In general a larger number of walks ensures a more accurate view on the subgraph area but this parameter has to be selected with respect to graph size and walk length since these will influence computational complexity. The results will vary based on the selected parameters and the structure of the investigated network. This implies that for shorter walks a lower number of walks will provide a sufficient overview over the subgraph area, while for longer walks more walks are needed to scan the subgraph area (due to the increase in walk possibilities), indicating that the results are more sensitive to changes in number of performed walks for longer walks in comparison to shorter ones. Since only two networks are compared a larger number of walks is performed than in the network clustering analysis in order to reduce computational time. For each starting node the visited nodes are counted, ranked and a Kendall rank correlation is estimated between the two networks for each gene. This has been calculated through the *example_pipeline_wrappers.get_walk_distances.helper_get_counts()* and *example_pipeline_wrappers.get_walk_distances.helper_walk_sim()* function. The genes with the lowest rank correlation values are shown in Table 6.

Table 6: Genes with lowest rank correlation based on random walks from the same starting node in both networks.

Entrez ID	Gene Symbol
10206	*TRIM13*
7832	*BTG2*
29937	*NENF*
8396	*PIP4K2B*
6856	*SYPL1*
11098	*PRSS23*
6845	*VAMP7*
23047	*PDS5B*
10921	*RNPS1*
10105	*PPIF*

Among all the genes with a low rank correlation identified through random walks, the first three, as shown in Table 6, are known to be relevant to a malignant phenotype. The E3 ubiquitin-protein ligase (TRIM13 product) is a regulator of both membrane

and secretory proteins turnover located onto the membrane of the Endoplasmic Reticulum (ER). This factor is involved in the alteration of the apoptotic process by mediating the proteasomal degradation of MDM2 and AKT1 proteasomal degradation. Tomar et al. [76], report that TRIM13 may act as a tumor suppressor. On the other hand, the antiproliferative protein BTG2 is a known cell cycle regulator, involved in the G1/S transition [77]. Finally, the NENF protein product is a neurotrophic factor involved in the growth and differentiation of neuronal progenitors [78].

After comparing the individual nodes (genes) between the two networks, we investigate the edges. The dasatinib network contains 10820 edges and the mitoxantrone network contains 15308 edges, of these edges only 750 are shared between the networks, indicating that the connectivity between the networks is highly different.

Communities

It is well known that in real networks the nodes are not randomly connected with each other. Rather, they are prone to form distinct clusters of nodes which are tightly connected within each other, rather than the rest of the network. Such structures are named communities or modules [79]. The same applies to molecular networks, where genes that are highly interconnected (and, therefore highly co-expressed) are likely to be, as well, co-regulated. Such a principle reflects a common functional involvement of genes belonging to the same community. For this reason, the identification of distinct gene communities within molecular networks (known as “community detection”) became a fundamental step of network analysis in order to functionally characterize such clusters of co-expressed genes.
In this case study, we performed a community detection in both of the networks by employing an ensemble of community detection algorithms, in order to increase the robustness of the identified communities. The employed algorithms are louvain [50], infomap [47] and label_propagation [49] as implemented in the community module. The consensus partitioning was estimated with the *communities.fast_consensus()* function. Through this analysis we identified 4 co-expression communities in the mitoxantrone network (min: 116, max: 349, median: 256) and 8 in the dasatinib one (min: 4, max: 215, median: 139.5). The community partitionings are depicted in Figure 17 and 18. Figure 19 shows the distance of the identified communities in both networks in terms of nodes content, calculated by the Jaccard distance. The overall similarity among communities is moderate-to-low, with the lowest distance between the only exception for the communities indicated as mitoxantrone_A549_1 and dasatinib_A549_1 communities (JID: 0.779XXX).

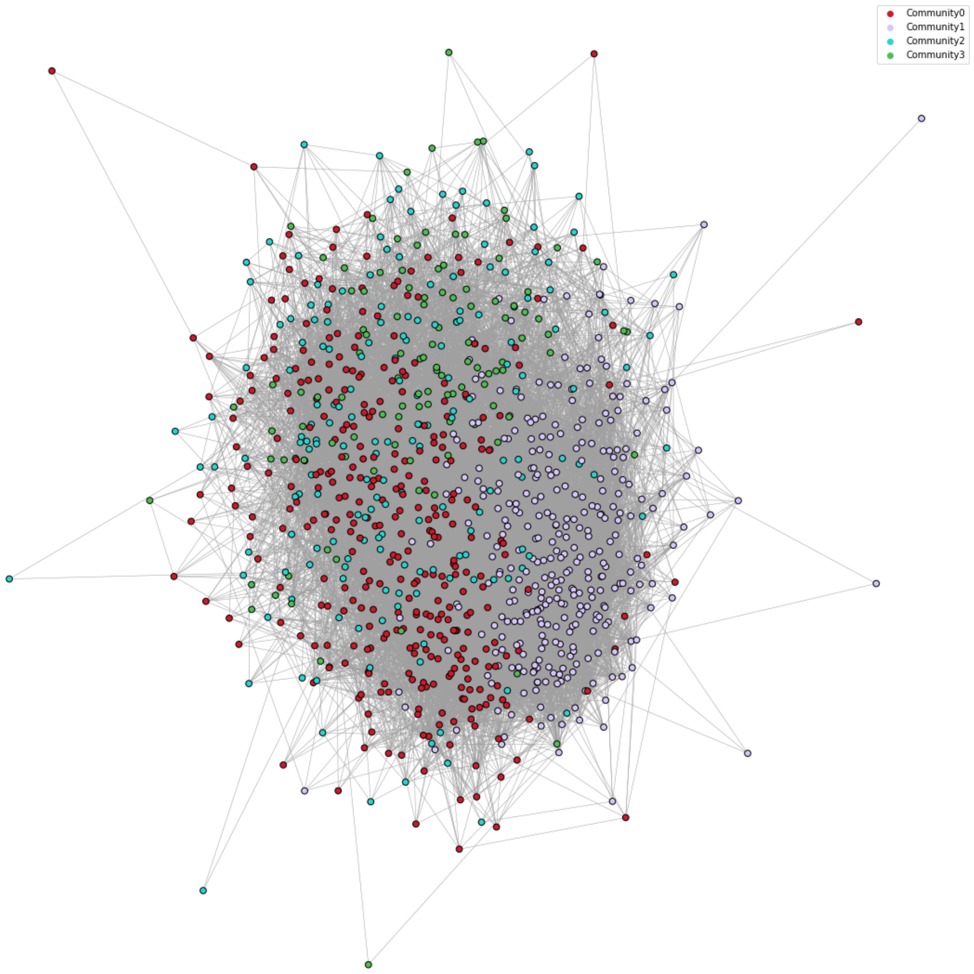

Figure 17: Partitioning of the mitoxantrone network.

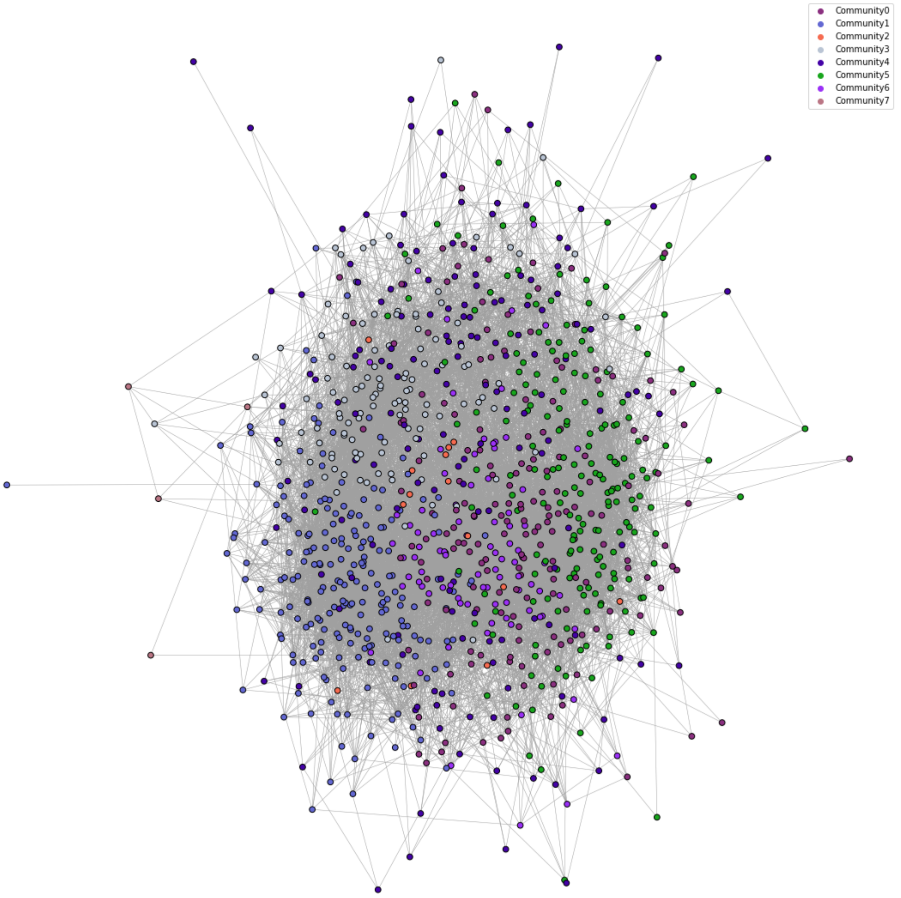

Figure 18: Partitioning of the dasatinib network.

This result indicates that the treatment with mitoxantrone and dasatinib has a different influence on the genes' co-expression patterns and that it might lead to a scarce functional resemblance, by deregulating distinct cellular processes. To verify this assumption, we evaluated the enrichment of Reactome pathways through the Panther enrichment API [56] with a Bonferroni correction and an applied p-value cutoff of <= 0.05.

The results of this analysis are summarized in Table 7. As expected, communities detected in the mitoxantrone network highlight the genotoxic effect of mitoxantrone by inducing DNA double strand breaks and by, in turn, deregulating the normal cell cycle activity. On the other hand, the functional characterization of the communities in the dasatinib network highlight the involvement of such a chemotherapeutic drug in the intracellular signaling processes. In fact, as a Tyrosine Kinase inhibitor, dasatinib is known to act by inhibiting peculiar mitogenic signaling cascades mediated by BCR-ABL, SRC family (SRC, LCK, YES, FYN), c-KIT, EPHA2, and PDGFRβ kinases [80]. Our analysis also showed that dasatinib might also induce the deregulation of genes belonging to the DNA damage recognition machinery.

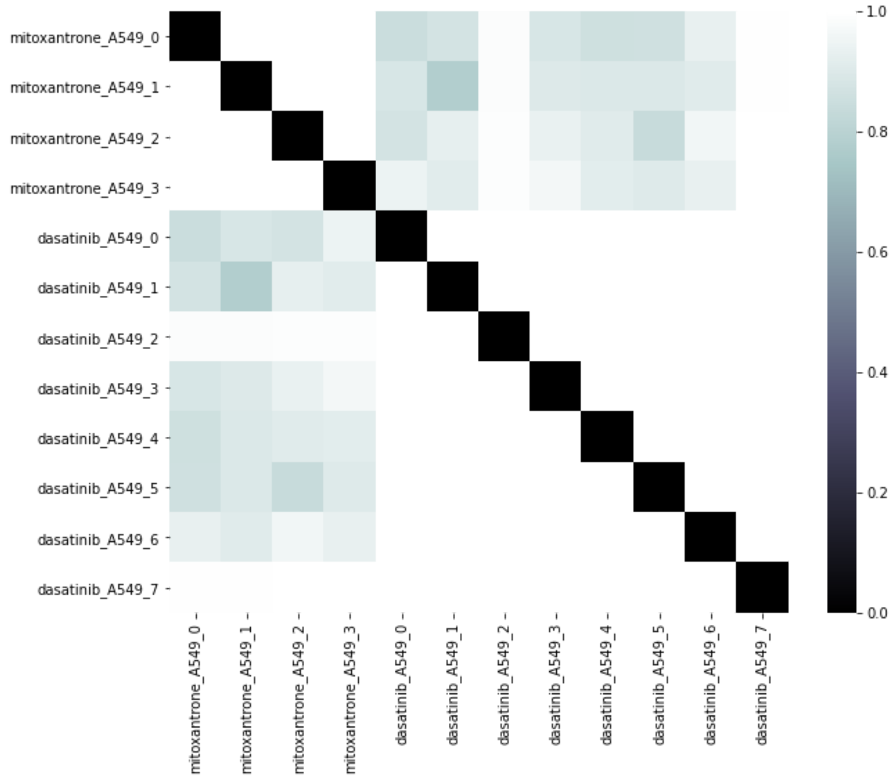

Figure 19: Jaccard Distance between the identified communities w.r.t. their nodes.

Table 7: Enriched pathways in the communities of the two networks.

Drug	Cluster	Enriched Reactome Pathway Term	P Value	FDR	Number in List	Number in Reference	Expected
Mitoxantrone	0	Immune System	0.003	0	74	2158	41.6
Mitoxantrone	0	DNA Double Strand Break Repair	0.005	0	14	148	2.9
Mitoxantrone	0	DNA Repair	0.011	0	20	309	5.96
Mitoxantrone	0	Cell Cycle Checkpoints	0.022	0	18	270	5.2
Mitoxantrone	0	G2/M Checkpoints	0.029	0	13	150	2.9
Mitoxantrone	1	Immune System	0.023	0	67	2158	38.7
Mitoxantrone	1	Metabolism of	0.037	0	62	1977	35.4

		proteins					
Mitoxantrone	2	Disease	0.032	0	27	1126	10.9
Mitoxantrone	2	Fc-gamma receptor (FCGR) dependent phagocytosis	0.046	0	9	149	1.4
Dasatinib	0	Immune System	0.046	0	43	2158	21.9
Dasatinib	1	Transcriptional regulation by RUNX2	0.006	0	10	117	1.4
Dasatinib	1	Disease	0.019	0	32	1126	13.6
Dasatinib	1	PTEN Regulation	0.023	0	10	137	1.7
Dasatinib	1	PIP3 activates AKT signaling	0.034	0	13	248	3
Dasatinib	1	Intracellular signaling by second messengers	0.035	0	14	287	3.5
Dasatinib	4	Recognition of DNA damage by PCNA-containing replication complex	0.001	0	6	30	0.3
Dasatinib	4	Resolution of AP sites via the multiple-nucleotide patch replacement pathway	0.011	0	5	25	0.2
Dasatinib	4	DNA Damage Bypass	0.013	0	6	48	0.4

4.3. Network Clustering

In order to compare the responses of different cell lines to the same drug treatment, we performed a clustering based on nodes, edges, structural properties as well as subgraph areas (Figure 20). The 20 networks investigated in this study consist of different cell lines as reported in Tables 4 & 6 and were treated with the same dose of dasatinib (10 um) at the same time point (24h).

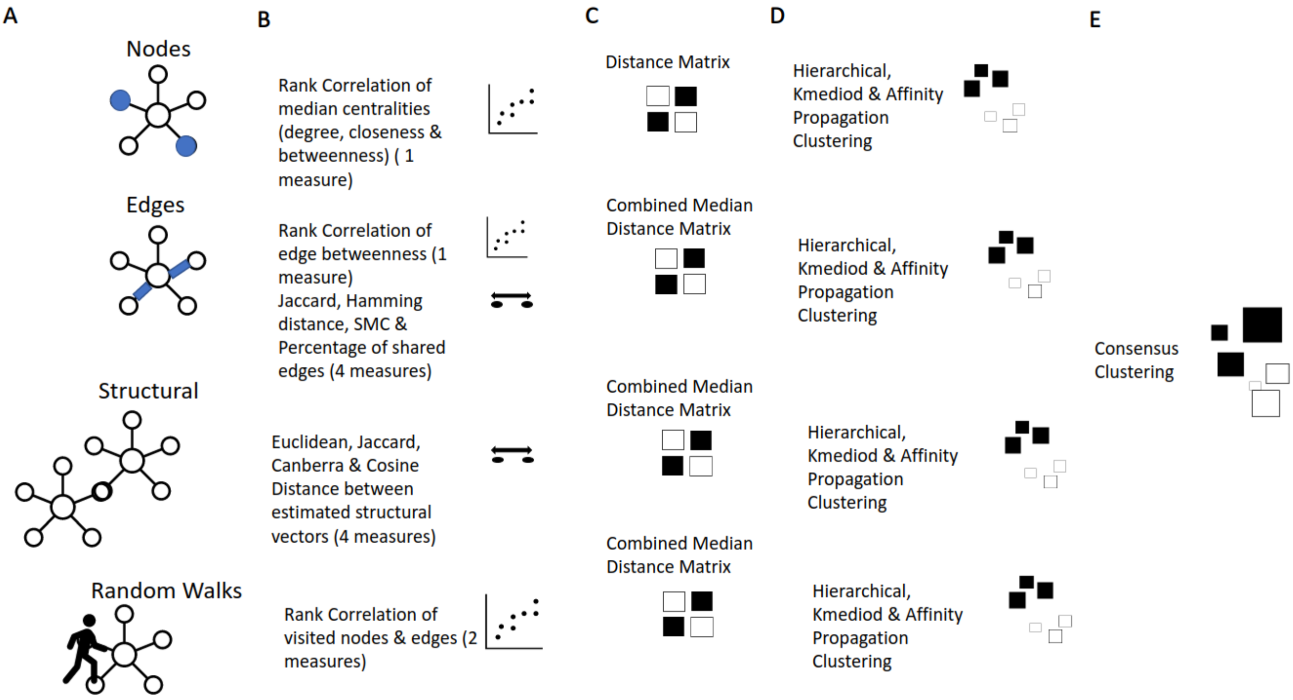

Figure 20: The networks are compared on four different aspects: nodes, edges, structural properties and subgraph areas by means of random walks (A). For each category different distance, similarity and correlation measures are calculated and transformed into distances (B). These measures are merged into a combined distance matrix (C). On the four individual distance matrices clustering is performed with three clustering algorithms (D). The individual clusterings are merged into a consensus clustering, yielding the final clustering (E).

First, the node rank positions (based on different centrality measures) between the networks are explored. With this kind of analysis, VOLTA allows to compare the networks based on the distribution of the centrality scores of their nodes. Degree-, closeness- and betweenness centrality as well as their mean and median node ranks are estimated with the *get_node_similarity.sort_list_and_get_shared()* function (Figure 20A). Based on these values the kendall rank correlation between all networks for the whole set of nodes is calculated with the *example_pipeline_warppers.get_node_similarity.estimate_similarities_nodes()* function. A correlation value for each of the individual centrality properties as well their mean and median is returned. The computed correlation values are in [-1,1], where a value of 1 indicates that the nodes are ranked in the same order, while a

value of -1 indicates the opposite. In this way, networks whose nodes are highly correlated are considered more similar between each other. The correlation matrix based on the median ranks of all three centrality measures is converted into a distance matrix (Figure 20B) via the transformation: *distance d = (1- correlation)/2*. In this way the correlation is translated into [0,1], where a value of 0 indicates that the nodes are ranked identical between two networks. This transformation is required since the later used clustering algorithms require distance matrices as input.
Correlation analysis is effective on networks that share a significant amount of nodes, by providing insight into a node's overall connectivity and importance in the network. However not all possible network types contain the same nodes, where the similarity between networks can be investigated based on their number of shared and unique nodes. Therefore this function additionally estimates the Jaccard, Hamming and SMC distances/similarity between the individual networks based on their nodes. However the investigated networks in this study all contain the same nodes,and therefore these metrics are omitted in this particular analysis.

Subsequently, edge similarities between the networks are investigated (Figure 20A). Since for this analysis unweighted networks are provided, edge weights are assigned to each edge based on their edge betweenness values. Then, shared edges and a binary edge representation for each network are estimated via the *example_pipeline_wrappers.get_edge_similarity.sort_list_and_get_shared()* function. Edge weights are not considered in this step. The output of this function output is given as input to the *example_pipeline_wrappers.get_edge_similarity.estimate_similarities_edges()* function, which returns the Jaccard, Hamming and SMC distance/ similarity between the existing edges of the networks as well as a percentage value of shared edges between each pair of input networks (Figure 20B). In contrast to the node comparison, where all networks shared the same nodes and therefore these distances were of low importance, they are significant when comparing the networks w.r.t. to their edges, since non complete networks are compared. This provides insight if the same nodes are connected through the same edges or not, which can provide insight into the underlying molecular interactions. Additionally, the Kendall rank correlation based on edge betweenness values of the top 100 edges is returned (this selection can differ between the networks) (Figure 20B). If weighted networks are provided the edges can be compared directly based on their edge weight rankings. The correlation values are again transformed into a distance with the same formula as used in the previous step. Additionally the similarity values are converted into a distance through the transformation: *distance d = 1 - similarity*. The four individual measurements are combined into a single distance matrix through the *clustering.create_median_distance_matrix()* function (Figure 20C). This is done so that the clustering at the end will receive the same amount of input distance matrices for each of the four categories (Figure 20A) on which a consensus clustering is performed.

As a next step, the topological/structural similarities between the networks are compared. For each network a feature vector, based on a variety of topological measures is computed with the *example_pipeline_wrappers.get_network_structural_vector.estimate_vector()* function (Figure 20A). This function computes the graph size (radius, diameter, number of nodes and number of edges), the density, the average clustering, the percentage of existing and non existing edges in comparison to a complete graph, number of cycles and their size (number of edges a cycle is made up of) distribution (mean, median, standard deviation, skewness and kurtosis of cycle size), shortest path distribution (mean, median, standard deviation, skewness and kurtosis), clustering coefficient, degree distribution (mean, median, standard deviation, skewness and kurtosis), closeness centrality distribution (mean, median, standard deviation, skewness and kurtosis), betweenness centrality distribution (mean, median, standard deviation, skewness and kurtosis). Between these vectors, the euclidean, canberra, correlation, cosine and jaccard distance are estimated with the *example_pipeline_wrappers.get_network_structural_vector.matrix_from_vector()* function (Figure 20B) and combined into a median distance matrix through the *clustering.create_median_distance_matrix()* function (Figure 20C).

The final category focuses on subgraph areas (Figure 20A). For each common node between every network pair (in the case of the investigated networks all nodes are shared), 3 times node degree random walks of length 5 are performed, with the *example_pipeline_wrappers.get_walk_distances.helper_walks()* function. By estimating the number of walks for each node based on its degree the subarea of each node is explored in a comparable fashion. A short walk length is selected in order to limit the area to be explored. These parameters can be selected by the user as needed. For each start node, the visited nodes and edges are counted, ranked on their occurrence and the Kendall rank correlation for the top 10 visited nodes and edges is estimated with the *example_pipeline_wrappers.get_walk_distances.helper_get_counts()* and *example_pipeline_wrappers.get_walk_distances.helper_walk_sim()* function between each network pair for the same starting node (Figure 20B). Again, these two measurements (nodes and edges) are transformed into a distance with the above transformation and combined into a single distance matrix (Figure 20C), as explained in the previous steps.

After the individual distance matrices of the four categories have been calculated, a consensus clustering is performed (Figure 20D). First, parameter optimization is run with the help of the *clustering.multiobjective()* function for the hierarchical clustering (*clustering.hierarchical_clustering()*) and k-medoids (*clustering.kmedoids_clustering()*) clustering algorithm. The multiobjective function has multiple parameters, a user can select, to perform parameter selection of clustering algorithms. Here we optimized to have high within cluster similarity, low between cluster similarity and an even cluster size distribution. Since the affinity

propagation algorithm (*clustering.affinityPropagation_clustering()*) does not contain any parameters, it is not included in the parameter optimization round. After the best parameters for each of the algorithms have been selected, each algorithm is run 10 times on each of the four created distance matrices. This is performed since some of the used clustering algorithms have randomness. Multiple runs of the same algorithm can therefore provide different clusterings, which are considered during the consensus clustering. The algorithms not containing randomness are run the same amount of times to ensure each algorithm is valued equally during the consensus clustering step. Based on the 120 individual clusterings, a consensus clustering is created (Figure 20E) through the *clustering.consensus_clustering()* function, its implementation is based on Brain Connectivity Toolbox for Python (https://github.com/aestrivex/bctpy). The *consensus_clustering* algorithm first constructs an agreement graph, where each network is represented as a node and an edge between two nodes is weighted by the fraction of clusterings these two nodes (networks) are clustered together. The function provides different options on how to prune the agreement graph, here it is set to “matrix”, which indicates a threshold (to identify weak edges) is estimated based on a permutation of the adjacency matrix. On the pruned graph community detection via the louvain [50] algorithm is performed (here 10 times) and the process is continued until a convergence is reached. Since the clustering algorithms as well as some of the distance estimation matrices contain a certain degree of randomness, the final results will vary slightly for each run.

As shown in Table 8, we obtained 3 distinct clusters (clusters 0, 1 and 2) of networks. Arguably, such clusters reflect both the tissue of origin and the disease status of the cell line. Cluster 1 mainly contains networks representing breast cancer cell lines, with the exception of MCF7 cells, falling into cluster 0. Malignant melanoma cell line A375 also belongs to the cluster 1. On the other hand, cluster 2 is populated by networks representing normal cell lines of different origin, spanning from the central nervous system to the adipose tissue. These results suggest that the tissue of origin, even across different cell lines, determines common patterns, which are particular of the tissue. As well, this is evident from the cellular status, since all of the networks belonging to cluster 2 represent non-transformed cell lines. This might highlight that such networks could contain patterns underlying physiological (or quasi-physiological) cellular processes. Such common graph patterns are explored in section 3.4.

Table 8: Clustering of the 20 Cell lines treated with dasatinib.

Cell Line treated with dasatinib	Assigned Cluster	Origin	Satus	Morphology

HEPG2	0	liver	hepatocellular carcinoma	epithelial-like
HCC515	0	lung	adenocarcino ma	epithelial
MCF7	0	breast	adenocarcino ma	epithelial
PC3	0	prostate	adenocarcino ma	epithelial
HA1E	0	kidney	normal	epithelial
HT29	0	colon	colorectal adenocarcino ma	epithelial
A549	0	lung	carcinoma	epithelial-like
MCF10A	1	breast	fibrocystic disease	epithelial
BT20	1	breast	carcinoma	epithelial
HS578T	1	breast	carcinoma	epithelial
A375	1	skin	malignant melanoma	epithelial
SKBR3	1	breast	adenocarcino ma	epithelial
MDAMB231	1	breast	adenocarcino ma	epithelial
NPC	2	central nervous system	normal	neural progenitor cells
NEU	2	central nervous system	normal	neuronal cells
SKL	2	skeletal muscle cells	normal	muscle cells
CD34	2	bone	normal	hematopoietic stem
ASC	2	adipose tissue	normal	fibroblast-like stem cells

HUVEC	2	vascular system/ umbilical cord	normal	endothelial
HME1	2	breast	normal	epithelial-like

4.4. Investigating a group of networks

In order to showcase VOLTA's capability to identify properties that characterize a set of graphs, the networks grouped in cluster 1 of Table 8 are investigated more closely. First, we look into which structures are statistically overrepresented in these networks. For each edge, its p-value based on the hypergeometric function as well as the Benjamini - Hochberg correction [55] is calculated with the *pattern_matching.get_statistical_overrepresented_edges()* function. Afterwards, all edges with an associated p-value of less or equal to 0.05 are selected and a new graph representing this cluster is constructed (1.4). This graph is depicted in Figure 21, plotted with the *plotting.plot_graph()* function.

The newly created graph is investigated for its most central genes. The top ten ranked nodes based on their median degree, closeness and betweenness ranking are shown in Table 9. Interestingly, some of the most central genes of the cluster 1 are involved in processes related to cell cycle, differentiation and metabolism. For instance, the *RAB21* gene belongs to the RAS oncogene family and it is involved in cell motility and adhesion processes. It is associated with glioma tumors and aberrant expression of this gene is associated with poor prognosis [81]. Discoidin Domain Receptor Tyrosine Kinase 1 (DDR1) gene product belongs to a subfamily of tyrosine kinase receptors. It is involved in the regulation of cell growth and differentiation and has been associated with a number of human cancers, including meningeal sarcoma and breast carcinoma [82].

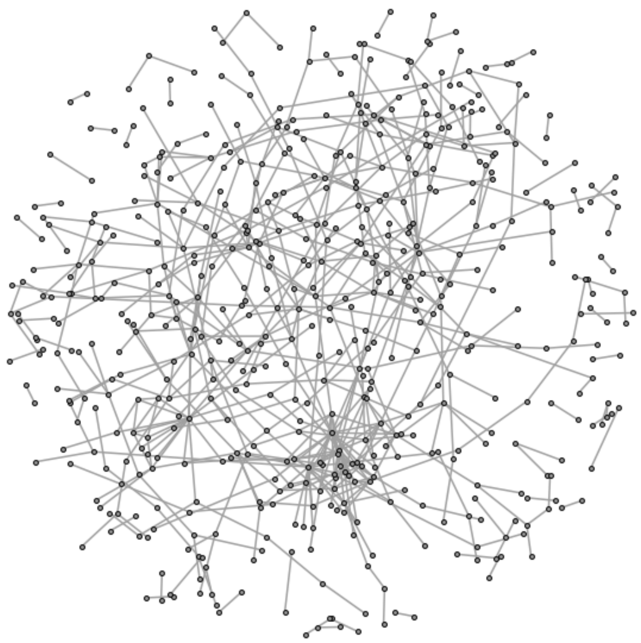

Figure 21: Network, made up of statistically significant edges overrepresented in cluster 1.

Table 9: The top most central nodes in the cluster 1 network, based on the median ranking of degree, closeness and betweenness centrality.

Entrez ID	Gene Symbol
5048	*PAFAH1B1*
23011	*RAB21*
6253	*RTN2*
10969	*EBNA1BP2*
780	*DDR1*
9761	*MLEC*
24138	*IFIT5*
1152	*CKB*
7398	*USP1*
23271	*FMO2*

For each network in cluster 1, the louvain community detection algorithm is run 10 times and a consensus clustering is created. This was computed with the *pattern_matching.get_consensus_community()* function. In total 4 communities (community 0-3) are detected of size 288, 149, 231 and 309. The communities are functionally enriched through the Panther enrichment API [56] against Reactome Pathways by applying a Bonferroni correction, of which two of the clusters enrich statistically significantly (p-value <= 0.05) for Reactome pathways. The enriched pathways are displayed in Table 10.

As shown in Table 10, the enriched terms on the communities 2 and 3 indicate a marked deregulation of immune-related pathways, together with cell cycle and DNA repair machinery. Indeed, while community 2 is enriched by genes involved in cytokine signaling and mitogenic signaling (M Phase and MAPK signaling pathway), community 3 is additionally enriched by genes involved in transcription-coupled nucleotide excision repair processes. These results indicate that communities 2 and 3 are characterized by a marked footprint of cell transformation together with the involvement of the immune compartment, arguably activated in response to such transformation and hyperproliferation, typical of malignant phenotypes.

Table 10: Community Enrichment of Reactome Pathways

Community	Enriched Reactome Pathway Term	PValue	Number in List	Number in Reference	Expected
2	Cytokine Signaling in Immune system	0.00009	32	823	10.5
2	M Phase	0.04	16	352	4.5
2	MAPK family signaling cascades	0.04	14	276	3.53
3	Immune System	0.000001	76	2158	36.0
3	Cytokine Signaling in Immune system	0.0002	37	823	13.7
3	DNA Repair	0.0003	21	309	5.16
3	Innate Immune System	0.002	42	1105	18.5
3	Transcription-Coupled Nucleotide Excision Repair	0.003	10	78	1.3
3	Gap-filling DNA repair synthesis and ligation in TC-NER	0.006	9	64	1.1
3	Dual incision in TC-NER	0.007	9	65	1.1
3	Signaling by Interleukins	0.008	23	447	7.5

3	Gene expression (Transcription)	0.008	49	1451	24.2
3	Signal Transduction	0.009	77	2728	45.6
3	Nucleotide Excision Repair	0.011	11	110	1.8
3	Base Excision Repair	0.012	9	70	1.2
3	Resolution of Abasic Sites (AP sites)	0.012	7	36	0.6
3	Hemostasis	0.05	28	669	11.2

Bibliography

[1] C. R. Harris *et al.*, “Array programming with NumPy.,” *Nature*, vol. 585, no. 7825, pp. 357–362, Sep. 2020, doi: 10.1038/s41586-020-2649-2.

[2] J. D. Hunter, “Matplotlib: A 2D Graphics Environment,” *Comput. Sci. Eng.*, vol. 9, no. 3, pp. 90–95, 2007, doi: 10.1109/MCSE.2007.55.

[3] S. Seabold and J. Perktold, “Statsmodels: Econometric and Statistical Modeling with Python,” in *Proceedings of the 9th Python in Science Conference*, 2010, pp. 92–96, doi: 10.25080/Majora-92bf1922-011.

[4] F. Pedregosa *et al.*, “Scikit-learn: Machine Learning in Python .,” *J Mach Learn Res*, vol. 12, no. 85, pp. 2825–2830, 2011.

[5] A. A. Hagberg, D. A. Schult, and P. J. Swart, “Exploring network structure, dynamics, and function using networkX,” presented at the SciPy2008, Aug. 2008.

[6] G. Rossetti, L. Milli, and R. Cazabet, “CDLIB: a python library to extract, compare and evaluate communities from complex networks,” *Appl. Netw. Sci.*, vol. 4, no. 1, p. 52, Dec. 2019, doi: 10.1007/s41109-019-0165-9.

[7] P. Virtanen *et al.*, “Author Correction: SciPy 1.0: fundamental algorithms for scientific computing in Python.,” *Nat. Methods*, vol. 17, no. 3, p. 352, 2020, doi: 10.1038/s41592-020-0772-5.

[8] P. Virtanen *et al.*, “SciPy 1.0: fundamental algorithms for scientific computing in Python.,” *Nat. Methods*, vol. 17, no. 3, pp. 261–272, Mar. 2020, doi: 10.1038/s41592-019-0686-2.

[9] V. A. Traag, L. Waltman, and N. J. van Eck, “From Louvain to Leiden: guaranteeing well-connected communities.,” *Sci. Rep.*, vol. 9, no. 1, p. 5233, Mar. 2019, doi: 10.1038/s41598-019-41695-z.

[10] M. Waskom *et al.*, “mwaskom/seaborn: v0.9.0 (July 2018),” *Zenodo*, 2018, doi: 10.5281/zenodo.592845.

[11] J. Reback *et al.*, “pandas-dev/pandas: Pandas 1.0.3,” *Zenodo*, 2020, doi: 10.5281/zenodo.3509134.

[12] W. McKinney, “Data structures for statistical computing in python,” in *Proceedings of the 9th Python in Science Conference*, 2010, pp. 56–61, doi: 10.25080/Majora-92bf1922-00a.

[13] S. M. Van Dongen, “Graph clustering by flow simulation.,” Doctoral dissertation, 2000.

[14] S. Behnel, R. Bradshaw, C. Citro, L. Dalcin, D. S. Seljebotn, and K. Smith, “Cython: The Best of Both Worlds,” *Comput. Sci. Eng.*, vol. 13, no. 2, pp. 31–39, Mar. 2011, doi: 10.1109/MCSE.2010.118.

[15] A. Novikov, “Pyclustering: data mining library,” *JOSS*, vol. 4, no. 36, p. 1230, Apr. 2019, doi: 10.21105/joss.01230.

[16] T. Aynaud, *python-louvain 0.13: Louvain algorithm for community detection.* GitHub, 2020.

[17] G. Rossetti, “Exorcising the Demon: Angel, Efficient Node-Centric Community Discovery,” in *Complex networks and their applications VIII*, vol. 881, H. Cherifi, S. Gaito, J. F. Mendes, E. Moro, and L. M. Rocha, Eds. Cham: Springer International Publishing, 2020, pp. 152–163.

[18] W.-M. Song *et al.*, “Network models of primary melanoma microenvironments identify key melanoma regulators underlying prognosis.,” *Nat. Commun.*, vol. 12, no. 1, p. 1214, Feb. 2021, doi: 10.1038/s41467-021-21457-0.

[19] M. A. Reyna *et al.*, “Pathway and network analysis of more than 2500 whole cancer genomes.,” *Nat. Commun.*, vol. 11, no. 1, p. 729, Feb. 2020, doi: 10.1038/s41467-020-14367-0.

[20] Y. Yang, L. Han, Y. Yuan, J. Li, N. Hei, and H. Liang, “Gene co-expression network analysis reveals common system-level properties of prognostic genes across cancer types.,” *Nat. Commun.*, vol. 5, p. 3231, 2014, doi: 10.1038/ncomms4231.

[21] Q. Zhang, J. E. Burdette, and J.-P. Wang, “Integrative network analysis of TCGA data for ovarian cancer.,” *BMC Syst. Biol.*, vol. 8, p. 1338, Dec. 2014, doi: 10.1186/s12918-014-0136-9.

[22] T. Huang *et al.*, “A network analysis of biomarkers for type 2 diabetes.,” *Diabetes*, vol. 68, no. 2, pp. 281–290, 2019, doi: 10.2337/db18-0892.

[23] M. Liu *et al.*, “Network-based analysis of affected biological processes in type 2 diabetes models.,” *PLoS Genet.*, vol. 3, no. 6, p. e96, Jun. 2007, doi: 10.1371/journal.pgen.0030096.

[24] J. Fernández-Tajes *et al.*, “Developing a network view of type 2 diabetes risk pathways through integration of genetic, genomic and functional data.,” *Genome Med.*, vol. 11, no. 1, p. 19, Mar. 2019, doi: 10.1186/s13073-019-0628-8.

[25] R. Ahn, R. Gupta, K. Lai, N. Chopra, S. T. Arron, and W. Liao, “Network analysis of psoriasis reveals biological pathways and roles for coding and long non-coding RNAs.,” *BMC Genomics*, vol. 17, no. 1, p. 841, Oct. 2016, doi: 10.1186/s12864-016-3188-y.

[26] E. Piruzian *et al.*, “Integrated network analysis of transcriptomic and proteomic data in psoriasis.,” *BMC Syst. Biol.*, vol. 4, p. 41, Apr. 2010, doi: 10.1186/1752-0509-4-41.

[27] P. Bertolazzi, M. E. Bock, and C. Guerra, “On the functional and structural characterization of hubs in protein-protein interaction networks.,” *Biotechnol. Adv.*, vol. 31, no. 2, pp. 274–286, Apr. 2013, doi: 10.1016/j.biotechadv.2012.12.002.

[28] T. Milenković and N. Pržulj, “Uncovering biological network function via graphlet degree signatures,” *Cancer Inform.*, vol. 6, p. CIN.S680, Jan. 2008, doi: 10.4137/CIN.S680.

[29] P. Kinaret *et al.*, “Network analysis reveals similar transcriptomic responses to intrinsic properties of carbon nanomaterials in vitro and in vivo.,” *ACS Nano*, vol. 11, no. 4, pp. 3786–3796, Apr. 2017, doi: 10.1021/acsnano.6b08650.

[30] J. Zhang *et al.*, “Comparison of gene co-networks reveals the molecular mechanisms

of the rice (Oryza sativa L.) response to Rhizoctonia solani AG1 IA infection.," *Funct. Integr. Genomics*, vol. 18, no. 5, pp. 545–557, Sep. 2018, doi: 10.1007/s10142-018-0607-y.

[31] M. Berlingerio, D. Koutra, T. Eliassi-Rad, and C. Faloutsos, "Netsimile: A scalable approach to size-independent network similarity," *arXiv preprint arXiv:1209.2684*, 2012.

[32] W. Hayes, K. Sun, and N. Pržulj, "Graphlet-based measures are suitable for biological network comparison.," *Bioinformatics*, vol. 29, no. 4, pp. 483–491, Feb. 2013, doi: 10.1093/bioinformatics/bts729.

[33] T. Milenković, W. L. Ng, W. Hayes, and N. Przulj, "Optimal network alignment with graphlet degree vectors.," *Cancer Inform.*, vol. 9, pp. 121–137, Jun. 2010, doi: 10.4137/cin.s4744.

[34] N. Przulj, "Biological network comparison using graphlet degree distribution.," *Bioinformatics*, vol. 23, no. 2, pp. e177-83, Jan. 2007, doi: 10.1093/bioinformatics/btl301.

[35] Y. Giarratano *et al.*, "A framework for the discovery of retinal biomarkers in optical coherence tomography angiography (OCTA)," in *Ophthalmic Medical Image Analysis: 7th International Workshop, OMIA 2020, Held in Conjunction with MICCAI 2020, Lima, Peru, October 8, 2020, Proceedings*, vol. 12069, H. Fu, M. K. Garvin, T. MacGillivray, Y. Xu, and Y. Zheng, Eds. Cham: Springer International Publishing, 2020, pp. 155–164.

[36] P. Jaccard, "Nouvelles Recherches Sur La Distribution Florale. ," *ulletin de la Société vaudoise des Sciences Naturelles,* vol. 44, pp. 223–270, 1908.

[37] R. W. Hamming, *Coding and Information Theory*. Englewood Cliffs, New Jersey: Prentice-Hall Inc., 1980.

[38] L. Hubert and P. Arabie, "Comparing partitions," *J. of Classification*, vol. 2, no. 1, pp. 193–218, Dec. 1985, doi: 10.1007/BF01908075.

[39] M. G. Kendall, "Kendall, Maurice G. "A new measure of rank correlation.," *Biometrika*, vol. 30, no. 1/2, pp. 81–93, 1938.

[40] E. Katifori and M. O. Magnasco, "Quantifying loopy network architectures.," *PLoS ONE*, vol. 7, no. 6, p. e37994, Jun. 2012, doi: 10.1371/journal.pone.0037994.

[41] C. D. Modes, M. O. Magnasco, and E. Katifori, "Extracting hidden hierarchies in 3D distribution networks," *Phys. Rev. X*, vol. 6, no. 3, p. 031009, Jul. 2016, doi: 10.1103/PhysRevX.6.031009.

[42] R. B. Tanvir and A. M. Mondal, "Cancer Biomarker Discovery from Gene Co-expression Networks Using Community Detection Methods," in *2019 IEEE International Conference on Bioinformatics and Biomedicine (BIBM)*, Nov. 2019, pp. 2097–2104, doi: 10.1109/BIBM47256.2019.8982960.

[43] S. Rahiminejad, M. R. Maurya, and S. Subramaniam, "Topological and functional comparison of community detection algorithms in biological networks.," *BMC Bioinformatics*, vol. 20, no. 1, p. 212, Apr. 2019, doi: 10.1186/s12859-019-2746-0.

[44] S. Zhang, "Comparisons of gene coexpression network modules in breast cancer and ovarian cancer.," *BMC Syst. Biol.*, vol. 12, no. Suppl 1, p. 8, Apr. 2018, doi: 10.1186/s12918-018-0530-9.

[45] V. S. Marwah *et al.*, "Inform: inference of network response modules.," *Bioinformatics*, vol. 34, no. 12, pp. 2136–2138, Jun. 2018, doi: 10.1093/bioinformatics/bty063.

[46] A. Tandon, A. Albeshri, V. Thayananthan, W. Alhalabi, and S. Fortunato, "Fast consensus clustering in complex networks.," *Phys. Rev. E*, vol. 99, no. 4–1, p. 042301,

Apr. 2019, doi: 10.1103/PhysRevE.99.042301.
[47] M. Rosvall and C. T. Bergstrom, “Maps of random walks on complex networks reveal community structure.,” *Proc Natl Acad Sci USA*, vol. 105, no. 4, pp. 1118–1123, Jan. 2008, doi: 10.1073/pnas.0706851105.
[48] P. Pons and M. Latapy, “Computing communities in large networks using random walks,” in *Computer and Information Sciences - ISCIS 2005*, vol. 3733, pInar Yolum, T. Güngör, F. Gürgen, and C. Özturan, Eds. Berlin, Heidelberg: Springer Berlin Heidelberg, 2005, pp. 284–293.
[49] U. N. Raghavan, R. Albert, and S. Kumara, “Near linear time algorithm to detect community structures in large-scale networks,” *Phys. Rev. E*, vol. 76, no. 3, Sep. 2007, doi: 10.1103/PhysRevE.76.036106.
[50] V. D. Blondel, J.-L. Guillaume, R. Lambiotte, and E. Lefebvre, “Fast unfolding of communities in large networks,” *J. Stat. Mech.*, vol. 2008, no. 10, p. P10008, Oct. 2008, doi: 10.1088/1742-5468/2008/10/P10008.
[51] F. Radicchi, C. Castellano, F. Cecconi, V. Loreto, and D. Parisi, “Defining and identifying communities in networks.,” *Proc Natl Acad Sci USA*, vol. 101, no. 9, pp. 2658–2663, Mar. 2004, doi: 10.1073/pnas.0400054101.
[52] J. Shi and J. Malik, “Normalized cuts and image segmentation,” *IEEE Trans. Pattern Anal. Mach. Intell.*, vol. 22, no. 8, pp. 888–905, 2000, doi: 10.1109/34.868688.
[53] G. W. Flake, S. Lawrence, and C. L. Giles, “Efficient identification of Web communities,” in *Proceedings of the sixth ACM SIGKDD international conference on Knowledge discovery and data mining - KDD ’00*, New York, New York, USA, Aug. 2000, pp. 150–160, doi: 10.1145/347090.347121.
[54] S. Fortunato, “Community detection in graphs.,” *Phys. Rep.*, vol. 486, no. 3–5, pp. 75–174, Feb. 2010, doi: 10.1016/j.physrep.2009.11.002.
[55] Y. Benjamini and Y. Hochberg, “Controlling the false discovery rate: A practical and powerful approach to multiple testing,” *Journal of the Royal Statistical Society: Series B (Methodological)*, vol. 57, no. 1, pp. 289–300, Jan. 1995, doi: 10.1111/j.2517-6161.1995.tb02031.x.
[56] H. Mi *et al.*, “Protocol Update for large-scale genome and gene function analysis with the PANTHER classification system (v.14.0).,” *Nat. Protoc.*, vol. 14, no. 3, pp. 703–721, Feb. 2019, doi: 10.1038/s41596-019-0128-8.
[57] H. Mi *et al.*, “PANTHER version 16: a revised family classification, tree-based classification tool, enhancer regions and extensive API.,” *Nucleic Acids Res.*, vol. 49, no. D1, pp. D394–D403, Jan. 2021, doi: 10.1093/nar/gkaa1106.
[58] G. Csardi and T. Nepusz, “The igraph software package for complex network research.,” *InterJournal, complex systems*, vol. 1695, no. 5, pp. 1–9, 2006.
[59] S. Proost and M. Mutwil, “CoNekT: an open-source framework for comparative genomic and transcriptomic network analyses.,” *Nucleic Acids Res.*, vol. 46, no. W1, pp. W133–W140, Jul. 2018, doi: 10.1093/nar/gky336.
[60] B. K. Kuntal, A. Dutta, and S. S. Mande, “CompNet: a GUI based tool for comparison of multiple biological interaction networks.,” *BMC Bioinformatics*, vol. 17, no. 1, p. 185, Apr. 2016, doi: 10.1186/s12859-016-1013-x.
[61] B. Boucher and S. Jenna, “Genetic interaction networks: better understand to better predict.,” *Front. Genet.*, vol. 4, p. 290, Dec. 2013, doi: 10.3389/fgene.2013.00290.
[62] S. J. Dixon, M. Costanzo, A. Baryshnikova, B. Andrews, and C. Boone, “Systematic mapping of genetic interaction networks.,” *Annu. Rev. Genet.*, vol. 43, pp. 601–625, 2009, doi: 10.1146/annurev.genet.39.073003.114751.

[63] H. Jeong, X. Qian, and B.-J. Yoon, “Effective comparative analysis of protein-protein interaction networks by measuring the steady-state network flow using a Markov model.,” *BMC Bioinformatics*, vol. 17, no. Suppl 13, p. 395, Oct. 2016, doi: 10.1186/s12859-016-1215-2.

[64] D. Hui, “Analysis of Human Metabolic Core Using Hub-Based Centrality,” in *2009 ETP International Conference on Future Computer and Communication*, Jun. 2009, pp. 88–90, doi: 10.1109/FCC.2009.18.

[65] P. Langfelder and S. Horvath, “WGCNA: an R package for weighted correlation network analysis.,” *BMC Bioinformatics*, vol. 9, p. 559, Dec. 2008, doi: 10.1186/1471-2105-9-559.

[66] V. C. Jardim, S. de S. Santos, A. Fujita, and M. S. Buckeridge, “Bionetstat: A tool for biological networks differential analysis.,” *Front. Genet.*, vol. 10, p. 594, Jun. 2019, doi: 10.3389/fgene.2019.00594.

[67] M. Fratello, A. Serra, V. Fortino, G. Raiconi, R. Tagliaferri, and D. Greco, “A multi-view genomic data simulator.,” *BMC Bioinformatics*, vol. 16, p. 151, May 2015, doi: 10.1186/s12859-015-0577-1.

[68] A. Subramanian *et al.*, “A next generation connectivity map: L1000 platform and the first 1,000,000 profiles.,” *Cell*, vol. 171, no. 6, pp. 1437-1452.e17, Nov. 2017, doi: 10.1016/j.cell.2017.10.049.

[69] J. J. Faith *et al.*, “Large-scale mapping and validation of Escherichia coli transcriptional regulation from a compendium of expression profiles.,” *PLoS Biol.*, vol. 5, no. 1, p. e8, Jan. 2007, doi: 10.1371/journal.pbio.0050008.

[70] A. A. Margolin *et al.*, “ARACNE: an algorithm for the reconstruction of gene regulatory networks in a mammalian cellular context.,” *BMC Bioinformatics*, vol. 7 Suppl 1, p. S7, Mar. 2006, doi: 10.1186/1471-2105-7-S1-S7.

[71] P. E. Meyer, K. Kontos, F. Lafitte, and G. Bontempi, “Information-theoretic inference of large transcriptional regulatory networks.,” *EURASIP J. Bioinform. Syst. Biol.*, p. 79879, 2007, doi: 10.1155/2007/79879.

[72] P. E. Meyer, F. Lafitte, and G. Bontempi, “minet: A R/Bioconductor package for inferring large transcriptional networks using mutual information.,” *BMC Bioinformatics*, vol. 9, p. 461, Oct. 2008, doi: 10.1186/1471-2105-9-461.

[73] L. G. Rossato *et al.*, “Mitochondrial cumulative damage induced by mitoxantrone: late onset cardiac energetic impairment.,” *Cardiovasc. Toxicol.*, vol. 14, no. 1, pp. 30–40, Mar. 2014, doi: 10.1007/s12012-013-9230-2.

[74] Y. Liao, J. Wang, E. J. Jaehnig, Z. Shi, and B. Zhang, “WebGestalt 2019: gene set analysis toolkit with revamped UIs and APIs.,” *Nucleic Acids Res.*, vol. 47, no. W1, pp. W199–W205, Jul. 2019, doi: 10.1093/nar/gkz401.

[75] B. Jassal *et al.*, “The Reactome Pathway Knowledgebase.,” *Nucleic Acids Res.*, vol. 48, no. D1, pp. D498–D503, Jan. 2020, doi: 10.1093/nar/gkz1031.

[76] D. Tomar, R. Singh, A. K. Singh, C. D. Pandya, and R. Singh, “TRIM13 regulates ER stress induced autophagy and clonogenic ability of the cells.,” *Biochim. Biophys. Acta*, vol. 1823, no. 2, pp. 316–326, Feb. 2012, doi: 10.1016/j.bbamcr.2011.11.015.

[77] B. Mao, Z. Zhang, and G. Wang, “BTG2: a rising star of tumor suppressors (review).,” *Int. J. Oncol.*, vol. 46, no. 2, pp. 459–464, Feb. 2015, doi: 10.3892/ijo.2014.2765.

[78] O. M. Koper-Lenkiewicz *et al.*, “Serum and cerebrospinal fluid Neudesin concentration and Neudesin Quotient as potential circulating biomarkers of a primary brain tumor.,” *BMC Cancer*, vol. 19, no. 1, p. 319, Apr. 2019, doi: 10.1186/s12885-019-5525-4.

[79] A.-L. Barabási and Z. N. Oltvai, “Network biology: understanding the cell’s functional

organization.," *Nat. Rev. Genet.*, vol. 5, no. 2, pp. 101–113, Feb. 2004, doi: 10.1038/nrg1272.

[80] A. E. Schade *et al.*, "Dasatinib, a small-molecule protein tyrosine kinase inhibitor, inhibits T-cell activation and proliferation.," *Blood*, vol. 111, no. 3, pp. 1366–1377, Feb. 2008, doi: 10.1182/blood-2007-04-084814.

[81] J. Ge, Q. Chen, B. Liu, L. Wang, S. Zhang, and B. Ji, "Knockdown of Rab21 inhibits proliferation and induces apoptosis in human glioma cells.," *Cell. Mol. Biol. Lett.*, vol. 22, p. 30, Dec. 2017, doi: 10.1186/s11658-017-0062-0.

[82] R. R. Valiathan, M. Marco, B. Leitinger, C. G. Kleer, and R. Fridman, "Discoidin domain receptor tyrosine kinases: new players in cancer progression.," *Cancer Metastasis Rev.*, vol. 31, no. 1–2, pp. 295–321, Jun. 2012, doi: 10.1007/s10555-012-9346-z.
